# Laser‐Induced Nanoscale Engineering of Iridium‐Based Nanoparticles for High‐Performance Oxygen Evolution

**DOI:** 10.1002/anie.202508589

**Published:** 2025-06-23

**Authors:** Huize Wang, Philipp Pfeifer, Wenwei Lai, Andreas Göpfert, Sumin Lim, Wei Zhao, A. Lucía Morales, Mattis Goßler, Marko Malinovic, Pallabi Bhuyan, Walter A. Parada, Pavlo Nikolaienko, Karl J. J. Mayrhofer, Guilherme V. Fortunato, Andreas Hutzler, Marc Ledendecker

**Affiliations:** ^1^ Forschungszentrum Jülich GmbH Helmholtz Institute Erlangen‐Nürnberg for Renewable Energy Cauerstraße 1 91058 Erlangen Germany; ^2^ Sustainable Energy Materials Technical University of Munich Campus Straubing Schulgasse 22 94315 Straubing Germany; ^3^ Department Chemical and Biological Engineering Friedrich‐Alexander‐Universität Erlangen‐Nürnberg Immerwahrstraße 2a 91058 Erlangen Germany

**Keywords:** Iridium oxide, Laser‐induced nano oven, OER, Solid‐state synthesis, Ultra‐small crystalline nanoparticles

## Abstract

While ruthenium oxide exhibits higher activity, it suffers from significantly lower stability in the acidic oxygen evolution reaction (OER). In contrast, crystalline iridium oxide is among the few materials that remain stable under such harsh conditions. However, its low activity and iridium scarcity require strategies to enhance atomic utilization. Conventional high‐temperature post‐synthetic processing increases the share of rutile‐phase iridium oxide while promoting particle growth, reducing catalytic activity due to a diminished surface area. Here, we present a laser‐induced nano‐oven method using a silicon dioxide matrix as a nanoscale reaction chamber, enabling solid‐state nanoparticle synthesis under ambient conditions while preventing agglomeration and allowing precise size control. The synthesized ultra‐small crystalline rutile iridium oxide of ∼2 nm achieves a high mass activity of 350 ± 15 A g_Ir_^−1^ at 300 mV overpotential, exceeding that of crystalline RuO₂ and reaching the activity benchmark of RuO_2_‐based catalysts. Analysis using a channel flow cell with on‐line inductively coupled plasma mass spectrometry (ICP‐MS) confirms that laser‐engineered iridium oxide exhibits superior stability to commercial iridium oxide. *Operando* electron impact MS provided the synthesis mechanistic insights, demonstrating the potential of this strategy for synthesizing ultra‐small crystalline metals and metal oxides for various applications.

## Graphical abstract

To address the stability‐activity trade‐off in acidic oxygen evolution reactions, a laser‐induced nano‐oven method is developed for the precise synthesis of ultra‐small crystalline iridium and iridium oxide nanoparticles under ambient conditions, employing a silicon dioxide matrix as a nanoscale reaction chamber. The synthesized ∼2 nm rutile IrO_2_ nanoparticles exhibit a high mass activity of 350 ± 15 A g_Ir_⁻¹ at 300 mV overpotential, achieving activity comparable to RuO₂ catalysts while demonstrating superior stability compared to commercial IrO_2_.

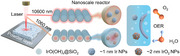

## Introduction

Nanoscale engineering plays a vital role in enhancing the activity and stability of catalysts, which are fundamental to driving advancements in electrochemical renewable energy generation and addressing environmental challenges.^[^ ^1^, ^2^ ^]^ Among various technologies, polymer electrolyte membrane water electrolysis (PEMWE) has emerged as a promising candidate to produce renewable hydrogen due to its ability to achieve high current densities, rapid dynamic response, and efficient hydrogen production under pressures exceeding 150 bar.^[^ ^3^, ^4^ ^]^ However, the sluggish kinetics of the oxygen evolution reaction (OER) at the anode, coupled with the extremely limited availability of catalysts capable of enduring the harsh acidic conditions, remain significant challenges.^[^ ^5^, ^6^ ^]^

Crystalline rutile‐phase IrO₂ demonstrates high dissolution stability, significantly surpassing other catalyst materials such as ruthenium oxide, amorphous iridium oxides, iridium‐based perovskites, and metallic iridium, despite the higher OER activity often demonstrated by the latter.^[^ ^7^, ^8^, ^9^ ^]^ Conventional high‐temperature post‐synthetic processing increases the share of rutile‐phase iridium oxide while promoting particle growth. This process results in enhanced stability due to the formation of robust iridium oxide structures but significantly reduces catalytic activity, as the increased particle size leads to a diminished surface area available for catalytic reactions.^[^ ^10^, ^11^ ^]^ Many approaches have been conducted to increase surface‐to‐volume ratios to achieve high activity, such as size control via encapsulation,^[^ ^12^ ^]^ core‐shell,^[^ ^13^ ^]^ porosity engineering,^[^ ^14^ ^]^ or supporting catalysts on high‐surface‐area materials.^[^ ^15^ ^]^ However, enhancing catalytic activity is frequently accompanied by a trade‐off in stability. While supporting strategies can indeed enhance catalytic activity, the dissolution‐induced degradation of active sites is ultimately determined by the intrinsic structural stability of iridium oxide.^[^ ^16^ ^]^ The preparation of ultra‐small, highly crystalline nanoparticles offers a promising solution for balancing activity and stability. The primary challenge is that high‐temperature, high‐energy methods typically employed to stabilize the rutile structure also induce particle growth.^[^ ^3^, ^12^, ^17^ ^]^ Therefore, developing innovative synthesis approaches to overcome this is urgently required.

The use of high‐energy focused laser beams for synthesizing metal‐based nanoparticles in liquid‐phase systems has gained significant attention in recent years.^[^ ^18^, ^19^ ^]^Although rapid cooling in liquid‐phase reactions enhances kinetic control over particle size, it reduces laser energy efficiency and photonic utilization. Solvents introduce significant complexities in nanoparticle purification, as their removal often requires time‐consuming and energy‐intensive steps such as evaporation, filtration, or centrifugation. Additionally, they pose challenges for large‐scale production due to their potential toxicity, environmental impact, and solvent‐particle interactions, all of which increase both costs and operational complexity. By contrast, solid‐phase reactions offer a classic, straightforward approach widely utilized in industrial synthesis.^[^ ^20^ ^]^

In this study, we introduce a laser‐induced solid‐state synthesis method for fabricating small crystalline Ir and IrO₂ nanoparticles in the size range of ca. 2 nm. Encapsulated iridium oxyhydroxide in SiO₂ was synthesized as a functional nano‐reactor, where the SiO₂ shell served as a protective and structural outer chamber. The encapsulation confined the iridium‐based material, while a high‐energy laser beam was utilized as a localized heating source to drive the thermal transformation and stabilization processes within the encapsulated structure. This design ensured controlled thermal processing while preserving the material's nanoscale features. Through X‐ray diffraction analysis and adjustments to laser power, scanning speed, and wavelength, we precisely controlled nanoparticle size and Ir oxidation states. To elucidate the synthesis mechanism, *operando* mass spectrometry (MS) was employed to monitor gas‐phase composition during laser irradiation, providing real‐time insights into reaction pathways. After screening, the optimized crystalline metallic Ir (∼1 nm) and rutile IrO₂ (∼2 nm) nanoparticles were assessed for OER activity and dissolution stability using an on‐line channel flow cell (CFC) integrated with inductively coupled plasma mass spectrometry (ICP‐MS).

## Results and Discussion

### Nanoscale Engineering by Laser‐Induced Solid‐State Synthesis

**Figure 1:**
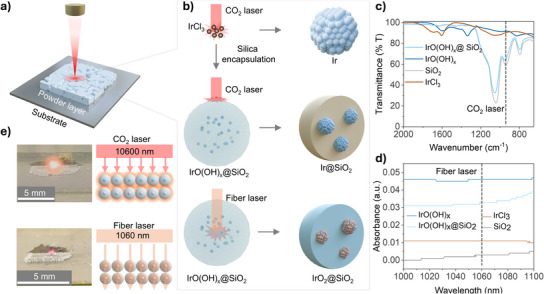
Schematic representation of the innovative synthesis concept based on a laser‐induced nano reactor design. a), b) Schematic representation illustrating the laser‐induced solid‐state synthesis of crystalline Ir and IrO₂ nanoparticles under ambient conditions. c) Infrared spectra of IrCl₃, SiO₂, IrO(OH)_X_, and IrO(OH)_X_@SiO₂ powders, highlighting absorption differences at 943 cm⁻¹, corresponding to the 10 600 nm wavelength of the CO₂ laser. d) UV–vis absorption spectra of IrCl₃, SiO₂, IrO(OH)_X_, and IrO(OH)_X_@SiO₂ in the 1000–1100 nm range. e) Photographs capturing the surface of the powder layer during irradiation with a CO₂ and a fiber laser.

In a first attempt, we explored the application of laser‐induced synthesized (Lis) iridium in the controlled fabrication of iridium‐based nanoparticles without an encapsulating matrix. When IrCl₃ was spread on a titanium (Ti) plate and irradiated with a CO₂ laser (λ = 10,600 nm) (Figure 1a,b), large iridium crystallites with sizes around 40 nm were formed, as determined by XRD (Figure S1).

To mitigate particle growth during laser treatment and achieve better size control, we encapsulated iridium oxyhydroxide within a SiO₂ shell, synthesized in a microemulsion (c.f. method section). This SiO₂ shell acted as a nanoscale confinement structure, preventing agglomeration while enabling precise control over particle formation. Importantly, the SiO₂ shell absorbed strongly at 943 cm⁻¹, corresponding to the 10,600 nm wavelength of the CO₂ laser, effectively acting as a localized nanoreactor to concentrate energy and drive the synthesis process (Figure 1c). In contrast, SiO₂ was nearly transparent at the 1,060 nm wavelength of a fiber laser (Figure 1d), which opens two opens two distinctly different laser treatment strategies (Figure 1b). Under CO₂ laser irradiation, the strong excitation of the Si─O─Si stretching vibration in the SiO₂ shell generated intense localized heating, driving the decomposition of IrO(OH)_X_ into metallic Ir. This process was accompanied by pronounced light emission, as captured in Figure 1e and Movie S1.

In contrast, the fiber laser, with its near transparency through the SiO₂ shell, directly targeted the IrO(OH)_X_ core. The weak absorption of IrO(OH)_X_ at 1,060 nm (Figure 1d) resulted in a more moderate and controlled thermal response. This facilitated the formation of iridium oxides rather than metallic Ir, as depicted in Figure 1e and Movie S2.

### Rapid Synthesis and Screening of Crystalline Nanostructured Catalysts

**Figure 2:**
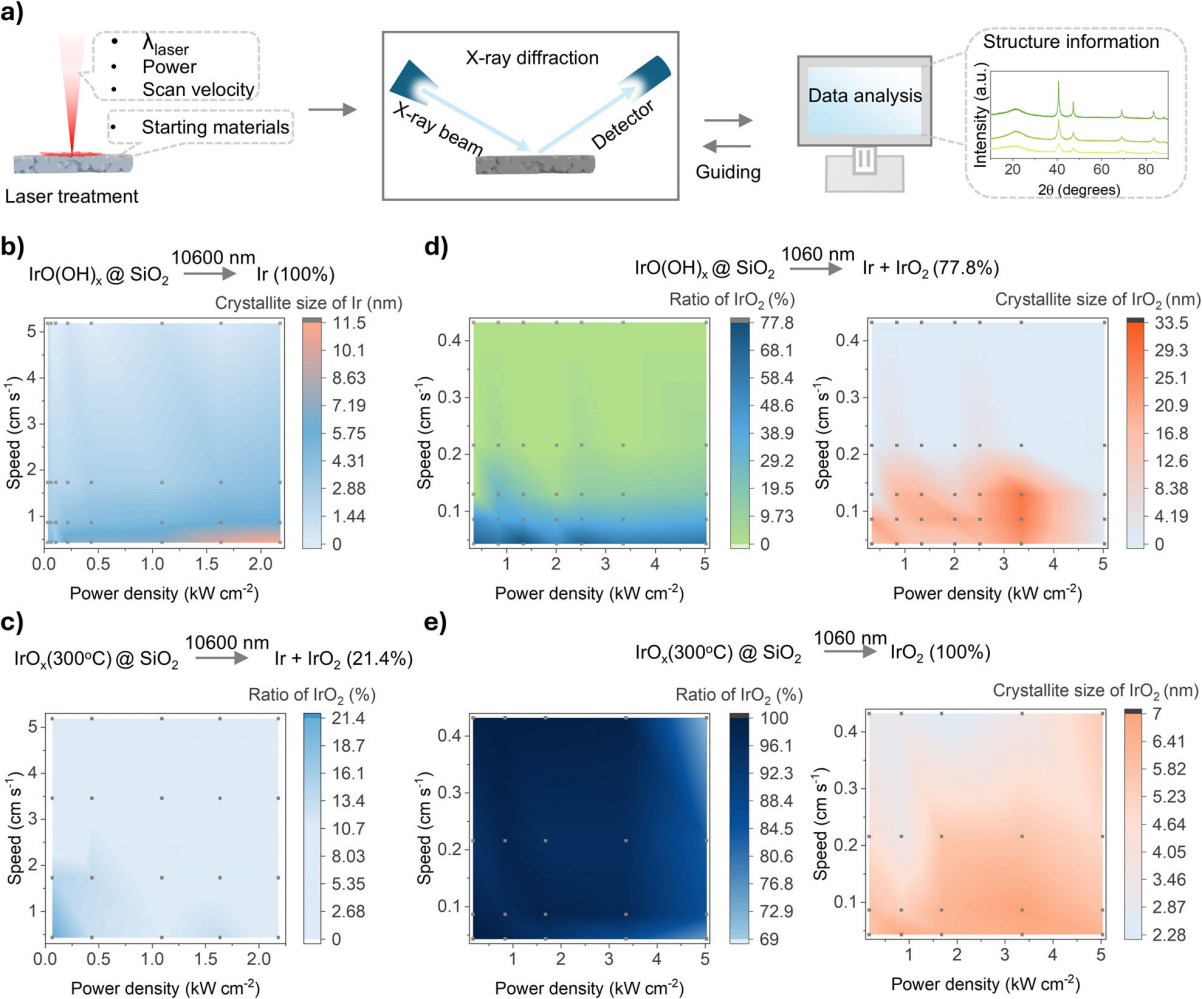
Screening and characterization of crystalline nanoparticle catalysts via XRD analysis. a) Schematic representation of the screening process for crystalline nanoparticle catalysts using XRD analysis, highlighting the influence of laser power, scanning speed, wavelength, and precursor materials. b) 2D plot showing the crystallite size of metallic Ir synthesized using IrO(OH)_X_@SiO₂ as the precursor, plotted against the power density and scanning speed of the CO₂ laser. c) 2D plot illustrating the ratio of crystalline IrO₂ produced using IrO_X_(300 °C) as the precursor, relative to power density and scanning speed of the CO₂ laser. d) 2D plot showing the ratio (left) and crystallite size (right) of crystalline IrO₂ synthesized using IrO(OH)_X_@SiO₂ as the precursor, plotted against power density and scanning speed of the fiber laser. e) 2D plot showing the ratio (left) and crystallite size (right) of crystalline IrO₂ synthesized using IrO_X_(300 °C) as the precursor, plotted against power density and scanning speed of the fiber laser. The spots in the figure are the laser parameters used in the experiment.

To fine‐tune the final material, various laser parameters were systematically adjusted, including power density, scanning speed, wavelength, and precursor selection, to tailor the properties of the synthesized nanomaterials, as illustrated in Figure 2. Structural insights, such as crystal size, composition, and crystallinity, were obtained via X‐ray diffraction (XRD) combined with Whole Powder Pattern Fitting (WPPF). These characterizations guided the optimization of synthesis conditions, enabling the precise design of catalyst materials with the desired properties.

In the initial experiments, a CO₂ laser was used to irradiate IrO(OH)_X_@SiO₂. Higher power densities and slower scanning speeds resulted in increased crystallite sizes (Figures 2b and S2–S5), suggesting that larger laser energy density promotes nanoparticle growth. However, the SiO₂ shell effectively constrained crystallite sizes to below ca. 12 nm. Despite using the lowest laser power, only metallic iridium was formed, indicating that the energy input was still excessive to enable the formation of alternative phases. To address this, IrO(OH)_X_@SiO₂ was preheated at 300 °C to reduce structural defects (by removing hydrogen and oxygen atoms), yielding an amorphous IrO_X_ phase (Figure S6). When this material was irradiated with a CO₂ laser at lower power densities, crystalline IrO₂ was observed in the XRD spectra (Figures 2c, S7 and S8), though its maximum yield was limited to 21%. This limitation is likely due to the strong absorption of SiO₂ and iridium oxide at 10,600 nm, causing a rapid temperature rise, potentially exceeding 1000 °C. At such high temperatures, iridium oxide thermally decomposes into metallic iridium.^[^ ^21^ ^]^

To synthesize pure IrO₂, we mitigated the high absorption of SiO₂ by using a fiber laser with a wavelength of 1060 nm. At this wavelength, SiO₂ is nearly transparent, allowing direct photon interaction with the core material (Figure 1d). This approach enabled a more controlled and moderate thermal response, as evidenced by the absence of strong light emission during the process (Figure 1e). By optimizing power density and scan rate, a significant increase in the proportion of crystalline IrO₂ was achieved compared to CO₂ laser treatments (Figures 2d and S9–S12). Using IrO_X_ pretreated at 300 °C (IrO*_x_* (300 °C)) as a precursor under specific fiber laser conditions, 100% crystalline rutile IrO₂ was successfully synthesized (Figures 2e, S13 and 14). Interestingly, the choice of precursor material strongly influenced the crystalline size of IrO₂. It is hypothesized that during laser‐induced decomposition of IrO(OH)_X_ to IrO₂, gas evolution damages the SiO₂ shell. This rupture facilitates the agglomeration of adjacent nanoparticles, leading to the formation of larger particles (Figure S15). In contrast, the preheated IrO_X_ at 300 °C minimized this effect, enabling better control over the crystallinity and particle size of the final material.

### Reaction Mechanism Analysis via *Operando* Mass Spectrometry

**Figure 3:**
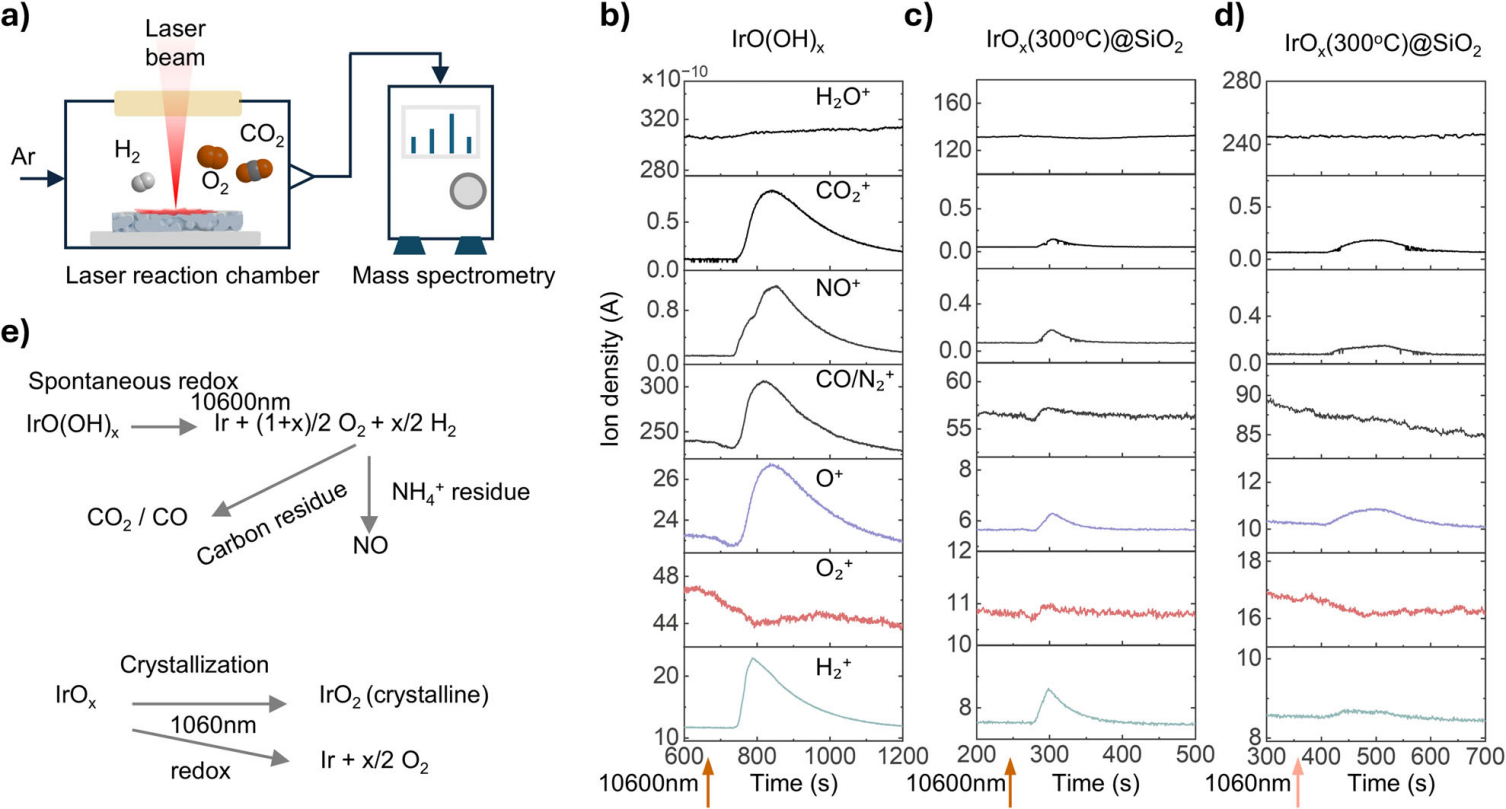
Real‐time reaction monitoring via *operando* MS. a) Schematic illustration of the *operando* MS setup for monitoring gas‐phase products generated by laser irradiation. MS spectra for H₂O^+^ (*m/z* = 18), CO₂^+^ (*m/z* = 44), NO^+^ (*m/z* = 30), CO/N₂^+^ (*m/z* = 28), O^+^ (*m/z* = 16), O₂^+^ (*m/z* = 32), and H₂^+^ (*m/z* = 2) are shown for: b) IrO(OH)_X_ as the starting material irradiated by a CO₂ laser at 0.8 W (1090 W cm^−2^) and a scanning speed of 0.216 cm s^−1^, c) IrO_X_(300 °C)@SiO_2_ as the starting material irradiated by a CO₂ laser at 1.6 W (2180 W cm^−2^) and a scanning speed of 5.184 cm s^−1^, d) IrO_X_(300 °C)@SiO_2_ as the starting material irradiated by a fiber laser at a power of 0.4 W (3354 W cm^−2^) and a scanning speed of 0.432 cm s^−1^. e) The CO₂ laser (λ = 10 600 nm) induces a spontaneous redox reaction, converting IrO(OH)_X_ into metallic Ir. In contrast, the fiber laser (λ = 1060 nm) facilitates the crystallization of amorphous IrO_X_, forming rutile crystalline IrO₂.

We employed a real‐time direct MS method to investigate reaction mechanisms to detect volatile compounds under specific laser conditions, offering insights into the activation energy for degradation and crystallization. As shown in Figure 3a, the laser beam passes through a gas‐tight chamber, with argon as the carrier gas flowing to a connected mass spectrometer.

As illustrated in Figure 3b, the treatment of IrO(OH)_X_ at 10600 nm resulted in substantial H₂ gas evolution. The ion density of oxygen‐containing gases such as CO₂^+^ (*m/z* = 44), NO^+^ (*m/z* = 30), and CO^+^ (*m/z* = 28) increased. In comparison, O₂^+^ (*m/z* = 32) initially decreased, likely due to the rapid generation of other gases, with a gradual increase afterward, suggesting that oxygen production occurs during preparation. Similar trends were noted with IrO(OH)_X_@SiO₂ (Figures S16 and S17); however, not many changes were observed with SiO₂ and this is attributed to the stability of Si─O bonds, as the laser energy arguably did not reach the ∼2000 °C required for thermal decomposition of SiO_2_.^[^ ^22^ ^]^ Instead, the observed signals likely originated from the dissociation of surface‐adsorbed substances. For IrO_X_(300 °C)@SiO₂ precursors, the attenuated hydrogen and oxygen signals compared to those of IrO(OH)_X_@SiO₂ indicated enhanced stability after the 300 °C preheating step (Figure 3c). Under fiber laser treatment, minimal oxygen or other gas signals were detected (Figure 3d), indicating limited dissociation. And combined with XRD analysis of solid‐phase components remaining after *operando* MS (Figure S17), an oxidizing atmosphere is required to ensure complete oxidation of iridium to iridium oxide.

Based on the MS and XRD results, we inferred the following mechanisms (Figure 3e):

(1) $$\mathrm{IrO}{\left(\mathrm{OH}\right)}_{x}\;\overset{10600\;\mathrm{nm}}{\to }\;\mathrm{Ir}+\left(1+x\right)/2\;O_{2}+x/2\;H_{2}$$

(2) $$\mathrm{Ir}O_{x}\left(\mathrm{amorphous}\right)\;\overset{1060\;\mathrm{nm}}{\to }\;\mathrm{Ir}O_{2}\left(\mathrm{crystalline}\right)$$

Under CO₂ laser irradiation, IrO(OH)*_X_* undergoes a spontaneous redox reaction, reducing Ir⁴⁺ to metallic Ir. The absence of detectable H₂O peaks suggests that hydroxyl groups and hydrates dissociate directly into H₂ and O₂. Oxygen further reacts with carbon residues to form CO₂ and CO. In contrast, fiber laser treatment provides a kinetically controlled approach to remove metastable oxygen from amorphous structures, enabling the structural transition to crystalline iridium oxide.

To elucidate the origin of the observed results, real‐time temperature monitoring was conducted using an infrared (IR) camera during the laser‐induced synthesis process. The results (Figures S18 and S19) indicate that the CO₂ laser irradiating the SiO₂ nanoscale chamber generates a higher temperature than the fiber laser acting on the core material, which facilitates the thermal decomposition of IrO(OH)_X_ and leads to the formation of metallic Ir. Given the limitations of the IR camera, the actual thermal profile is likely underestimated and warrants further investigation.

### Structure Characterization of the Optimized Catalyst

**Figure 4:**
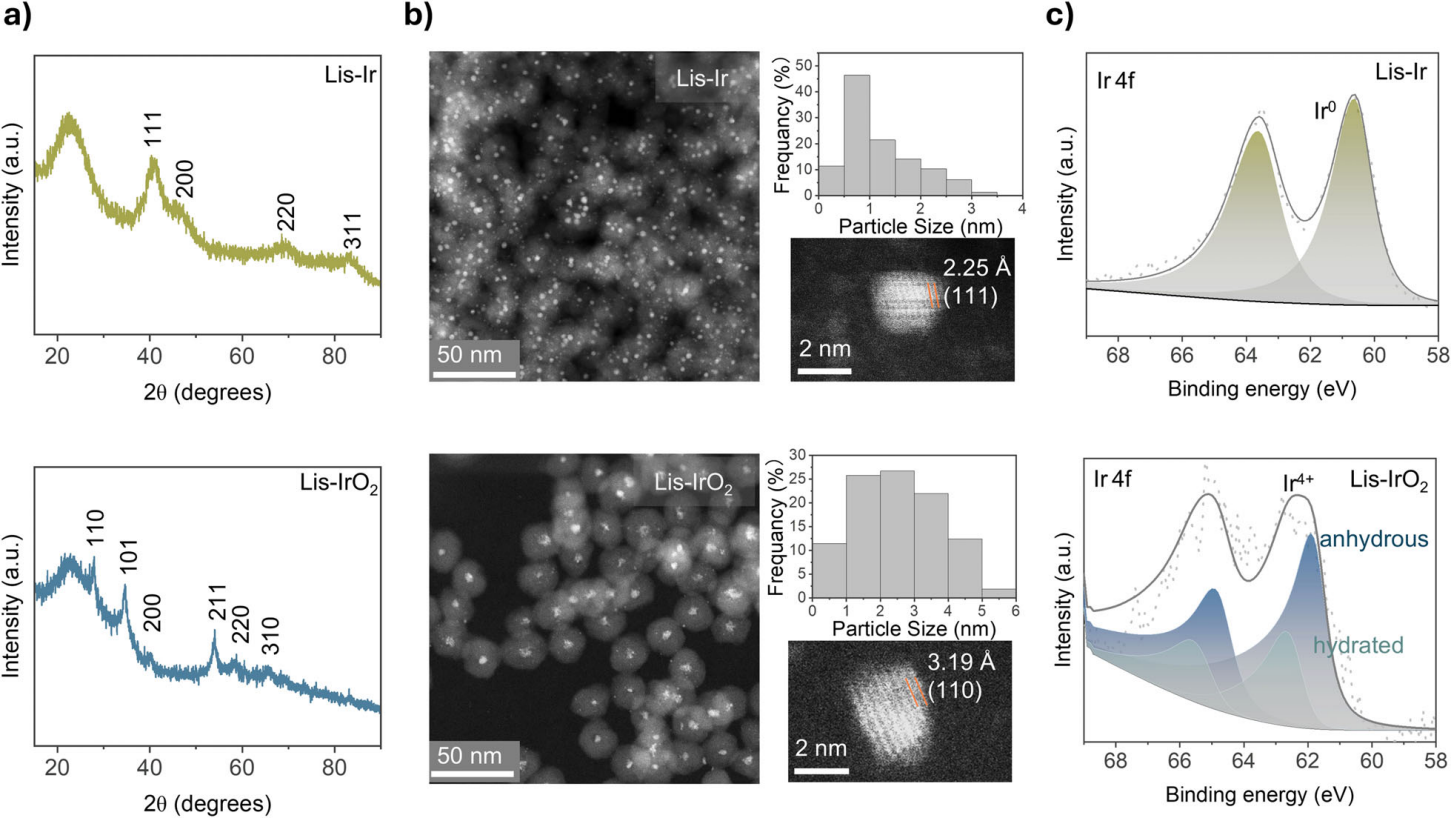
Structure characterization of ultra‐small crystalline Ir and IrO_2_ nanoparticles. a) XRD spectra of optimized catalysts Lis‐Ir and Lis‐IrO₂. Lis‐Ir was synthesized using IrO(OH)_X_@SiO₂ as a precursor, with a CO₂ laser power density of 32 W cm^−^^2^ and a scanning speed of 5.2 cm s^−1^. Lis‐IrO₂ was synthesized using IrO_X_(300 °C)@SiO₂ as a precursor, with a fiber laser power density of 1.67 kW cm^−^^2^ and a scanning speed of 0.432 cm s^−1^. b) HAADF‐STEM images of Lis‐Ir and Lis‐IrO₂, accompanied by particle size distribution histograms. c) XPS spectra of the Ir 4f core level for Lis‐Ir and Lis‐IrO₂.

Following catalyst screening and preliminary XRD characterization (Figure 4a), we selected two catalysts with small crystallite size and high crystallinity for further structural and electrochemical characterization in acidic OER conditions. The first catalyst, referred to as laser‐induced synthesized iridium (Lis‐Ir), was prepared using IrO(OH)_X_@SiO₂ as a precursor and a CO₂ laser. XRD analysis confirmed the formation of metallic Ir, with diffraction peaks corresponding to the (111), (200), (220), and (311) planes. Fitting of the diffraction peaks, based on the Scherrer equation, revealed a crystallite size of 1.3 nm. The second catalyst, referred to as laser‐induced synthesized IrO₂ (Lis‐IrO₂), was synthesized using IrO_X_(300 °C)@SiO_2_ as a precursor and a fiber laser. XRD analysis identified diffraction peaks corresponding to the (110), (101), (200), (211), (220), and (310) planes, characteristic of rutile IrO₂. Fitting analysis determined a crystallite size of 2.3 nm.

High‐angle annular dark‐field scanning transmission electron microscopy (HAADF‐STEM) was used to investigate the size and morphology of Lis‐Ir and Lis‐IrO₂ nanoparticles (Figures 4b, S20, and S21). The results show that both Ir and IrO₂ are evenly distributed within the SiO₂ spheres. Notably, neither CO₂ laser nor fiber laser treatment damaged the SiO₂ shell, as the spheres remained intact compared to untreated samples (Figure S20). For Lis‐Ir, particle size analysis revealed that most clusters were smaller than 1 nm, with crystalline metallic iridium particles predominantly in the 1–2 nm range. The TEM lattice fringes revealed a (111) lattice plane orientation with a measured fringe spacing of 2.25 Å consistent with the expected interplanar distance. For Lis‐IrO₂, the particle sizes were primarily distributed between 2–3 nm, with an average size of 2.5 ± 1.2 nm, aligning well with the XRD analysis. The observed rutile (110) lattice plane further confirmed the material's high crystallinity. X‐ray photoelectron spectroscopy (XPS) was employed to evaluate the chemical state of iridium in Lis‐Ir and Lis‐IrO₂ (Figure 4c). In the Ir 4f region of Lis‐Ir, peaks at 60.7 eV (Ir 4f₇/₂) and 63.7 eV (Ir 4f₅/₂) correspond to Ir(0).^[^ ^23^ ^]^ For Lis‐IrO₂, the primary peaks at 61.8 eV (Ir 4f₇/₂) and 64.8 eV (Ir 4f₅/₂) correspond to anhydrous IrO₂, while the peaks at 62.5 eV (Ir 4f₇/₂) and 65.5 eV (Ir 4f₅/₂), associated with hydrated or amorphous iridium oxide, are reduced compared to the precursor IrO_X_(300 °C)@SiO₂ (Figure S22).^[^ ^24^ ^]^ These results confirm that fiber laser irradiation facilitates structural rearrangement within the material.

### OER Performance of Laser‐Induced Synthesized Catalysts

**Figure 5:**
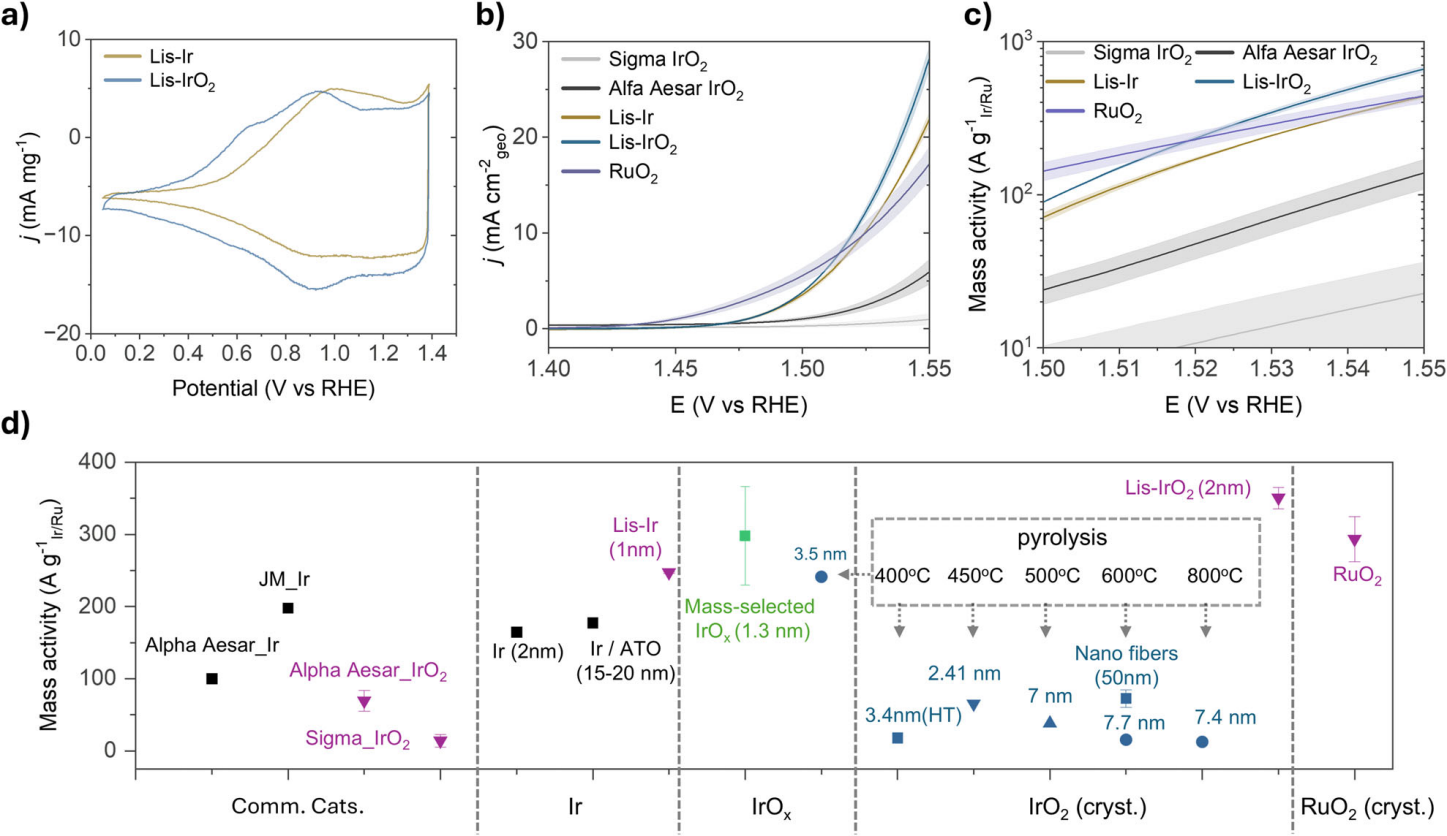
OER activity of laser‐induced synthesized Ir and IrO_2_. a) Mass normalized cyclic voltammetry measured with a scan rate of 50 mV s^−1^ between 0.05 and 1.39 V_RHE_. b) Polarization curves of Lis‐Ir, Lis‐IrO₂, Alfa Aesar IrO₂, Sigma IrO₂, and RuO_2_ catalysts in 0.1 M HClO₄ electrolyte. The geometric current density and standard error for laser‐induced synthesized and reference catalysts are based on multiple samples. c) Mass‐normalized activities of Lis‐Ir, Lis‐IrO₂ and three reference catalysts, Alfa Aesar IrO₂, Sigma IrO₂, and RuO_2_. d) Comparison of the mass activity of laser‐induced synthesized catalysts with other state‐of‐the‐art Ir‐based OER catalysts in acidic media at 1.53 V_RHE_. The average mass activity and standard error for laser‐induced synthesized catalysts and commercial IrO_2_ are based on multiple samples. Reference data were extracted from the literature: Alfa Aesar Ir (Ref. [37]), Johnson Matthey Ir nanoparticle (JM_Ir) (Ref. [38]), 2 nm Ir nanoparticle (Ref. [39]), antimony‐doped tin oxide supported Ir nanodendrites (Ir/ATO) (Ref. [25]), mass‐selected IrO*_x_* (1.3 nm) (Ref. [31]), 3.5 nm IrO*_x_* (heated at 400 °C) (Ref. [12]), 3.4 nm hydrothermal synthesized IrO_2_ (heated at 400 °C) (Ref. [40]), 2.41 nm nanoporous IrO_2_ (heated at 450 °C) (Ref. [41]), 7 nm IrO_2_ (heated at 500 °C)(Ref. [42]), 7.7 nm crystalline IrO_2_ (heated at 600 °C) (Ref. [12]), IrO_2_‐based nanofibers (heated at 600 °C) (Ref. [43]), 7.4 nm crystalline IrO_2_ (heated at 800 °C) (Ref. [12]).

To evaluate the OER performance of Lis‐Ir and Lis‐IrO₂, the SiO₂ shell was first removed as described in detail in the Methods. Three reference catalysts were used for comparison: Alfa Aesar IrO₂, which exhibits an amorphous oxide structure (Figure S23); Sigma IrO₂, which displays a highly crystalline rutile structure; and RuO₂ obtained by annealing at 600 °C for 3 h, showing a crystallite size of 19 nm according to XRD analysis (Figure S24). Electrochemical tests were conducted in 0.1 M HClO₄. In the cyclic voltammogram (CV) of Lis‐Ir, pronounced Ir^3^⁺/Ir⁴⁺ redox peaks were observed within the 0.6–1.2 V range (Figure 5a), indicating the formation of hydrous iridium oxide species and the loss of metallic Ir character after potential cycling, consistent with previous reports.^[^ ^25^, ^26^ ^]^ Consistent with XPS analysis, the CV profile of Lis‐IrO₂ resembles that of hydrous iridium oxide, lacking the characteristic double‐layer capacitance observed in anhydrous IrO₂ (Figure S25).^[^ ^12^, ^27^ ^]^ As shown in Figure 5a,b, Lis‐IrO₂ exhibited the highest activity in the linear sweep voltammetry (LSV) test compared to Lis‐Ir and the reference IrO₂ catalysts. Furthermore, Lis‐IrO₂ exhibited a lower overpotential of 289 mV at a current density of 10 mA cm⁻^2^, compared to 296 mV for RuO₂, which falls within the range reported in previous studies.^[^ ^28^, ^29^ ^]^

Tafel analysis (Figure S25) reveals that Lis‐IrO₂ exhibits the highest intrinsic kinetic activity, with a Tafel slope of 38.7 mV dec⁻¹. In addition, the electrochemically active surface area (ECSA) was estimated by integrating the Ir redox charge from cyclic voltammetry data collected between 0.4 and 1.3 V vs. RHE. As summarized in Table S1, although the commercial Alfa Aesar IrO₂ exhibits a higher ECSA due to its amorphous structure, Lis‐IrO₂ displays significantly higher specific activity and turnover frequency (TOF). Electrochemical impedance spectroscopy (EIS) analysis (Figure S26) further confirms that both Lis‐IrO₂ and Lis‐Ir exhibit lower charge‐transfer resistance, reflecting more favorable interfacial reaction kinetics, which contributes to their enhanced OER activity.

Figure 5c presents a detailed comparison of Lis‐IrO_2_ with commercial IrO₂ and other reported Ir‐based acidic OER catalysts. Lis‐IrO₂ exhibits an application‐relevant mass activity of 350 ± 15 A g_Ir_^−1^ at 1.53 V_RHE_, significantly outperforming all previously reported crystalline IrO₂ catalysts. This includes catalysts with small nanoparticles synthesized via high‐temperature methods, as well as commercial IrO₂ references, with enhancements ranging from 4‐ to 20‐fold. For comparison, Zhang et al. reported a mass‐selected cluster synthesis method that produced sub‐2 nm IrO₂ clusters with comparable activity. However, this approach is highly time‐ and cost‐intensive and is limited to nanogram‐scale loadings, restricting its practical applicability.^[^ ^30^, ^31^ ^]^ Similarly, Lis‐Ir demonstrates a mass activity of 247 ± 1 A g_Ir_^−1^at 1.53 V_RHE_, exceeding other ultra‐small Ir nanoparticle catalysts. Notably, both Lis‐Ir and Lis‐IrO₂ exhibit high catalytic activities comparable to that of RuO₂.

To gain initial insight into the structural stability of the rutile phase in Lis‐IrO₂, a 24‐h chronoamperometric (CA) durability test was conducted (Figure S27). Following the test, TEM analysis was performed to assess the morphological and structural evolution of electrocatalysts before and after electrochemical operation. As shown in Figure S28, for Lis‐Ir, pronounced particle agglomeration was observed. The initially dispersed sub‐nanoclusters were no longer present, and the material transformed into aggregated particles with sizes in the range of 2–3 nm. Nonetheless, lattice fringes corresponding to crystalline domains remain discernible, suggesting that partial crystallinity is retained. In contrast, Lis‐IrO₂ exhibited minimal morphological changes compared to the pristine sample. The particle size remained stable, and a majority of the nanoparticles preserved their well‐defined rutile crystalline structure.

**Figure 6:**
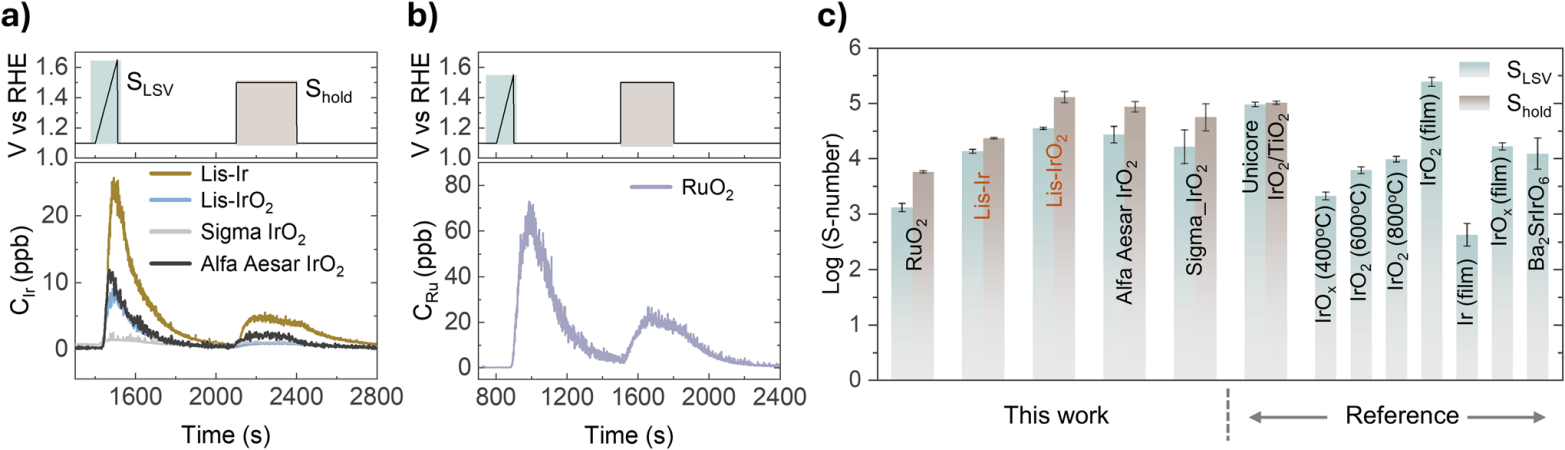
OER stability of laser‐induced synthesized Ir and IrO_2._ a) Dissolution stability analysis of laser‐induced synthesized catalysts and reference catalysts using CFC‐ICP‐MS. Potential profiles and measured Ir concentrations in the electrolyte were recorded during potential ramping to 1.65 V_RHE_ and under constant current conditions: 100 mA mg⁻¹_Ir_ for Lis‐Ir, Lis‐IrO₂, and Alfa Aesar IrO₂, and 20 mA mg⁻¹_Ir_ for Sigma IrO₂. b) Dissolution stability analysis of RuO_2_. Potential profiles and measured Ru concentrations in the electrolyte were recorded during potential ramping to 1.55 V_RHE_ and under constant current conditions of 100 mA mg⁻¹_Ru_. c) S‐number comparison of laser‐induced synthesized catalysts, reference catalysts, and other reported Ir‐based catalysts. S_LSV_ represents the S‐number obtained from LSV ramping, while S_hold_ corresponds to the S‐number measured after 5 min under constant current conditions. Reported catalysts include Umicore IrO₂/TiO₂ (S_LSV_ obtained from LSV ramping to 1.65 V_RHE_, S_hold_ at 20 mA mg⁻¹_Ir_)^[^ ^13^ ^]^; IrOx (400 °C), IrO₂ (600 °C), and IrO₂ (800 °C) (S_LSV_ obtained from LSV ramping to 5 mA cm⁻^2^, ∼1.62 V_RHE_)^[^ ^12^ ^]^; and IrO₂ (film), Ir (film), IrOx (film), and Ba₂SrIrO₆ (S_LSV_ obtained from LSV ramping to 1.65 VRHE).^[^ ^7^ ^]^

However, the interpretation of these results is complicated by the accumulation of microbubbles during prolonged CA measurements,^[^ ^32^, ^33^ ^]^ which can obscure meaningful comparisons of catalyst stability. Given that catalyst dissolution is widely recognized as the primary degradation mechanism in acidic OER,^[^ ^11^ ^]^ we have employed a CFC coupled with an ICP‐MS for real‐time monitoring of dissolution stability in laser‐synthesized catalysts. The measurement protocol consisted of linear sweeps from 1.1 to 1.65 V_RHE_ at 5 mV s^−1^ and thereafter inducing iridium‐normalized constant current densities of 100 mA mg^−1^ for Lis‐Ir, Lis‐IrO_2_, and Alfa Aesar IrO_2_ and 20 mA mg^−1^ for the Sigma IrO_2_ catalyst. Figures 6a,b, S29, and S30 illustrate the dissolution of iridium and ruthenium with the applied potential profiles for each catalyst separately. Although annealing can enhance the rutile phase content of RuO₂ and thereby improve its stability toward the OER,^[^ ^34^] as observed, the dissolution of RuO_2_ was highest during the first LSV scan and constant current density, exceeding that of Lis‐IrO_2_ by over an order of magnitude. In comparison with Lis‐IrO_2_ and reference IrO_2_ catalysts, Lis‐Ir exhibited greater dissolution and lower stability. This finding is consistent with previous reports.^[^ ^7^, ^35^ ^]^

Thereafter, we calculated the stability number (S‐number), which serves as a key metric for assessing catalyst stability. The S‐number is defined as the ratio of oxygen produced to dissolved iridium.^[^ ^7^ ^]^ The calculated S‐numbers are presented in Figure 6c. It is important to note that we have limited our comparisons to catalysts evaluated under comparable and similar experimental parameters owing to the sensitivity of S‐number values to changes in electrochemical testing procedures.^[^ ^36^ ^]^ The testing conditions for comparing catalysts are summarized in Table S2. It could be observed from Figure 6c that the Lis‐IrO₂ exhibits the highest S‐number among all the tested catalysts. Moreover, its stability progressively improved on imposing constant current density, ultimately demonstrating dissolution stability comparable to that of the most stable commercial catalyst, TiO₂‐supported crystalline IrO₂ nanoparticles from Umicore, which has lower activity.^[^ ^13^ ^]^ Although Lis‐IrO₂ does not match the stability of crystalline films, both Lis‐IrO₂ and Lis‐Ir exhibit mass activities that are several orders of magnitude higher than those of crystalline films.^[^ ^7^ ^]^ The observed stability is strongly influenced by the catalyst structure and surface species.^[^ ^35^ ^]^ These stability tests further confirm that Lis‐IrO₂ possesses a highly ordered crystalline structure, resulting in superior stability compared to metallic Ir, amorphous oxides, and hydrous oxides.

## Conclusion

In summary, we established a high‐energy laser‐based synthesis method enabling the direct, solid‐state fabrication of ultra‐small crystalline nanoparticles with precise size control. Using a fiber laser, we achieved precise kinetic control over the crystallization process to synthesize ∼2 nm rutile IrO₂ nanoparticles, which demonstrated enhanced catalytic activity and stability for acidic OER compared to previously reported crystalline IrO₂ catalysts and crystalline RuO_2_. Furthermore, CO₂ laser excitation facilitated efficient photon‐to‐thermal energy conversion, enabling the rapid synthesis of ∼1 nm crystalline metallic Ir nanoparticles with substantially lower energy requirements compared to conventional high‐temperature methods. The outstanding catalytic performance of the 2 nm rutile IrO₂ nanoparticles is attributed to their optimized particle size, which enhances active surface area, and a highly ordered crystalline structure that promotes stability under operating conditions. This methodology offers an energy‐efficient approach for developing low‐cost, corrosion‐resistant metal oxide catalysts tailored for acidic OER, while enabling precise kinetic control over metal oxidation states. Furthermore, it holds promise for synthesizing ultra‐small mixed metal and mixed metal oxide nanoparticles, with broad applicability across various electrocatalytic energy conversion processes.

## Author Contributions

H.W. and M.L. conceived and supervised the overall project. H.W. designed the experiments. H.W., W.L., A.G., and M.G. synthesized and characterized the catalysts. P.P., S.L., and W.Z. performed the catalytic performance evaluation. L.M. and A.H. conducted the HAADF‐STEM measurements. All authors contributed to the overall scientific interpretation and writing of the original draft.

## Conflict of Interests

The authors declare no conflict of interest.

## Supporting information



Suppporting Information

Suppporting Information

Suppporting Information

## Data Availability

The data that support the findings of this study are available from the corresponding author upon reasonable request.
