# Supplementary material for: Laser‐Induced Nanoscale Engineering of Iridium‐Based Nanoparticles for High‐Performance Oxygen Evolution
Source: Angew Chem Int Ed Engl. 2025 Jun 23;64(33):e202508589. doi: 10.1002/anie.202508589 (PMC12338381; doi:10.1002/anie.202508589)
Supplement: Supplementary file 1 — Suppporting Information [file ANIE-64-e202508589-s003.pdf]

Supporting Information

Laser-Induced Nanoscale Engineering of Iridium-Based Nanoparticles for High-Performance Oxygen Evolution

Huize Wang[1,2*], Philipp Pfeifer[2], Wenwei Lai[3], Andreas Göpfert[1,2], Sumin Lim[2], Wei Zhao[2], A. Lucía Morales[1,3], Mattis Goßler[1,2], Marko Malinovic[2], Pallabi Bhuyan[2], Walter A. Parada[1,3], Pavlo Nikolaienko[1], Karl J. J. Mayrhofer[1,3], Guilherme V. Fortunato[2], Andreas Hutzler[1], Marc Ledendecker[1,2*].

*Corresponding author: huiz.wang@fz-juelich.de; marc.ledendecker@tum.de

Table of Contents

Methods 3

Figure S1-S10 8

Figure S11-S20 18

Figure S21-S30 28

Figure S31 36

Table S1-S4 37

Supplementary References 41

Methods

Chemicals and materials

Iridium chloride hydrate (99.9%, Sigma-Aldrich), Ruthenium (III) chloride hydrate (99.9% Alfa Aesar), Sodium hydroxide (pellets, Sima-Aldrich), n-Heptane (>99%, Supelco), water (D.I., 18.2 MΩ·cm), Brij L4 (Mn ≈ 362, Sigma-Aldrich), Ammonia (32%, Supelco), Tetraethyl Orthosilicate TEOS (>99%, Sigma-Aldrich), Methanol, Acetone (99%+, Fisher Chemical), ethanol (absolute, ≥99 %, Fisher Scientific), hydrofluoric acid (48 %, Sigma-Aldrich).

Synthesis of $IrO(OH)_x@SiO_2$

$IrO(OH)_x@SiO_2$ was prepared according to a previously published protocol.[12] Before reverse microemulsion, the iridium precursor was hydrolyzed in a 10 ml vial. This was achieved by mixing 57.3 mg (0.181 mmol) of iridium chloride hydrate, 4.3 ml of ultra-pure water, and 0.7 ml of ammonia solution (32%). The resulting clear, brownish solution was stirred for 24 h at 400 rpm under ambient conditions. The solution turned purple, indicating the iridium oxidation state changed from III to IV. In a 500 ml round bottom flask, 27 ml Brij L4 was mixed with 120 ml n-heptane and stirred for 10 min. Afterward, the iridium (IV) containing aqueous solution was filtered and added dropwise to the round bottom flask, resulting in a reverse microemulsion. After 2 h stirring at 400 rpm in contact with air, 0.7 ml of ammonia solution (32%) was added to maintain a pH value of 10-11. Subsequently, 0.75 ml of TEOS (3.36 mmol) was introduced to encapsulate the formed iridium oxyhydroxide while stirring for additional 16 h (overnight). The core-shell particles were then precipitated by adding 150 ml of methanol, stirring for 5 min, and subsequent sedimentation of the solid for half an hour. The upper n-heptane phase was decanted. The methanol and nanoparticle-rich phases were centrifuged and washed with methanol and acetone, respectively. The obtained solid was dried under a vacuum at 60 °C in preparation for further experiments.

Synthesis of IrO_x(300 °C)@SiO_2

$IrO(OH)_x@SiO_2$ was heated in a tubular furnace under open environment with a temperature ramp of 2°C min^{-1}, and 3h of annealing time at temperatures of 300 °C.

Synthesis of reference RuO_2

0.2 M $RuCl_3 \cdot xH_2O$ was dissolved in 4 mL of ultra-pure water, followed by the dropwise addition of 4 mL of 1 M NaOH under continuous stirring for 3 hours. A black precipitate formed at the end of the reaction and was subsequently washed at least three times with deionized water to remove unreacted species. The resulting solid was then subjected to thermal treatment in a tubular furnace under ambient atmosphere, with a heating rate of 2 °C min^{-1} and an annealing time of 3 hours at 600 °C, yielding crystalline RuO_2.

Laser-induced synthesis

A high-precision laser engraving system (Speedy 400 flexx, Trotec) was utilized, integrating a 10,600 nm CO_2 laser and a 1,060 nm fiber laser. The CO_2 laser operated in pulsed mode at a

frequency of 10,000 Hz, while the fiber laser was set at 20,000 Hz. The scanning speed (v), originally expressed as a percentage, was converted to cm s^{-1}, and the power, also given as a percentage, was converted to watts (W). The resulting power density per unit area (W cm^{-2}) was calculated as the ratio of average power (W) to the beam area (cm^2). All power parameters used in this study are provided in Table S1-2.

During laser treatment, the powder precursor material is uniformly spread onto a titanium sheet. The powder layer area is adjustable as needed, and the laser scanning area is designed accordingly. The powder layer has a thickness of 0.2 mm (Figure S24), and the laser pattern consists of parallel line segments with an inter-line spacing of 0.2 mm.

XRD analysis

Rigaku SmartLab SE was used with a wavelength of Cu Kα = 1.54059 Å. Measurements were taken in a 2-theta range from 10° to 90° with a measurement speed of 1.5°/min using a background-free silicon sample holder.

The software SmartLab Studio II (Rigaku) was used to assist with XRD data analysis. Whole Powder Pattern Fitting (WPPF) was used to determine phase fractions, with peak profile fitting based on the Split Pseudo-Voigt (SPV) function.[1] Which is a purely empirical model, providing a precisely mathematical fit for peak shapes without explicitly accounting for physical phenomena like instrument or sample effects. Function as follows:

$$I(x) = \eta \cdot L(x) + (1 - \eta) \cdot G(x)$$

With $L(x)$: Lorentzian component, $G(x)$: Gaussian component, η: mixing parameter.

With the FP method, on the other hand, a physical model, factors contributing to peak broadening are accounted, such as: instrumental broadening (calibrated by standard samples) and sample broadening (grain size, micro-strain etc.), function as follows:

$$\beta_{obs}^2 = \beta_{instrument}^2 + \beta_{sample}^2$$

With β_{obs}: Observed Full Width at Half Maximum (FWHM), $\beta_{instrument}$: Instrumental broadening, β_{sample}: Sample broadening.

Under FP method profile fitting, the crystallite size D can be calculated with Scherrer equation:

$$D = \frac{K\lambda}{\beta_{sample} cos\theta}$$

With K: Scherrer constant (usually 0.89-1.0), λ: Wavelength of the X-ray. β_{sample}: Sample broadening, θ: Bragg's angle.

Furthermore, the fraction between the crystal phases is calculated as followed equation:

$$w_i(\%) = \frac{S_i M_i Z_i / V_i}{\sum_j S_j M_j Z_j / V_j} \times 100\%$$

Here S_i: Scale factor of crystal phase i or j, M_i: Formula units per cell in crystal phase i or j, Z_i: Molecular weight of the corresponding formula unit. V_i : cell volume of the crystal phase I or j.

Operando mass spectroscopy

Mass spectroscopy (Pfeiffer Omni Star GSD 320 Gas Analysis System) was used as an online detection of the change of gas components during the laser treatment on the precursors. The MS system was coupled to the concealed laser reaction chamber (Figure 3a) via silica tube, A ZnSe optical window was used for the CO_2 laser, while a fused silica window was used for the fiber laser, allowing the laser beam to pass through and irradiate the sample. Meanwhile, an inert gas stream carried volatile species from the reactor to the MS, ensuring efficient collection and detection.

The MS was baked out at 110 °C for 24 h in advance to remove the absorbed residue in the vacuum chamber of MS for better detection of low concentration gas components. Online gas monitor was operated under Selected Ion Monitoring (SEM) mode with Multiple Ion Detection (MID) at 1120V with inlet pressure of the capillary stabilized under around 3×10^{-6} mbar and working temperature of 90°C. The monitored m/z values included H_2O^+ (*m/z* = 18), CO_2^+ (*m/z* = 44), NO^+ (*m/z* = 30), CO/N_2^+ (*m/z* = 28), O^+ (*m/z* = 16), O_2^+ (*m/z* = 32), and H_2^+ (*m/z* = 2) corresponding to possible reaction products and intermediates from precursor thermal decomposition and laser-induced phase transformations. The scanning interval was set to 50 milliseconds per cycle to ensure high temporal resolution. Before the reaction started, MS ran with the inert gas flow for about 30 min to make sure the baselines of signals were all stabilized. Then the laser started, after about 5 to 10 minutes, the rise of multiple signals was observed, the laser induced synthesis reaction last for about 5 to 20 minutes. After the reaction, it continued running for another 20 minutes to ensure complete gas detection.

HAADF-STEM Measurements

HAADF-STEM characterization of synthesized nanomaterials was conducted using a Thermo Fischer Scientific Talos F200i operated at an acceleration voltage of 200 kV. The nanomaterials were treated with remote air plasma for 1 min using a PIE Scientific Tergeo EM plasma cleaner. Energy-dispersive X-ray spectroscopy (STEM-EDXS) recorded with a Dual Bruker XFlash 6|100 EDS detector further complemented qualitative elemental composition.

XPS Analysis

X-ray photoelectron spectroscopy (XPS) measurements were carried out on a Quantera II (Physical Electronics, Chanhassen, MN, USA) device, equipped with a monochromatic Al Kα-X-ray source (1486.6 eV). The X-ray source was operated at 15 kV and 25 W, leading to a circular spotsize with 100 μm diameter. Automatic charge neutralization was used during all measurements. Narrow spectra at Pd 3d and Ir 4f core levels were measured with a pass energy of 112 eV while the narrow spectra at O 1s and C 1s core levels as well as the survey spectra were acquired using a pass energy of 55 eV.

ATR-FTIR spectroscopy

Background-corrected infrared spectra of IrCl3, $IrO(OH)_x$, SiO_2, and IrO(OH)x@SiO_2 were recorded with a Perkin Elmar FTIR-Spectrometer Spectrum 3. The powdered samples were pressed on a ZnSe crystal and measured within a range of 4000 to 650 cm^{-1}.

UV-Vis spectroscopy

The absorbance of $IrCl_3$, $IrO(OH)_x$, and SiO_2 was measured to investigate the fiber laser mechanism. In the case of IrCl3, an aqueous solution with water was used as a reference. IrO(OH)x and SiO_2 nanoparticles formed during the reverse microemulsion were measured with a blank reverse microemulsion as a reference. The measurements were performed at a Shimadzu UV-1900i using a glass cuvette with a path length of 10 mm. The spectra were recorded within a range of 1000 to 1100 nm within 2 mins.

Laser Scanning Microscopes

The thickness of the powder layer on the titanium sheet was determined using confocal laser microscopy (VK-X1100, KEYENCE). The titanium sheet was used as the reference plane for profile analysis. The height-profile was measured along 4 vertical and 4 horizontal lines to check uniformity. An example microscope-image and height profiles are shown in Figure S26.

Catalyst evaluation

RDE Measurements:

The material needs to remove the silica shell via hydrofluoric acid to proceed with the next RDE measurements. For the removal of the silica shell, 75 mg of either Lis-Ir@SiO_2 or Lis-IrO_2@SiO_2 are placed in a 50 mL LDPE bottle, and 7.5 mL absolute ethanol and 275 µL 48% hydrofluoric acid are added. The bottle is closed, placed in an orbital shaker, and mixed for 24 h, to allow the complete etching of SiO_2. On the next day, the solutions were further diluted with about 20 mL ethanol and transferred into a centrifugation vial. The particles are washed two times with absolute ethanol before a final washing step with water. The catalyst material is collected and dried at 60 °C.

The catalysts were dispersed in an ink solution containing 80% (v/v) ultrapure water and 20% (v/v) isopropanol using an ultrasonication horn (Branson Sonifier) for 30 min. A volume of the dispersed ink was then drop-cast on a glassy carbon rotating disk electrode (RDE) and allowed to dry in air, creating a film with a catalyst loading of 0.05 mg cm^{-2}. The catalysts from Sigma Aldrich, Premion, Lis-Ir, and Lis-IrO_2 were used directly for measurements.

Electrochemical half-cell measurements were performed using a multi-necked glass flask. The experimental setup consisted of a three-electrode configuration: the catalyst films on a glassy carbon RDE as the working electrode, a graphite rod as the counter electrode, and a saturated Ag/AgCl electrode as the reference electrode. The electrolyte was 0.1 M $HClO_4$ (Rotipuran Ultra 70%, Carl Roth GmbH). The electrochemical measurements were conducted using a Gamry Reference 600 potentiostat and an Origalys OrigaTrod rotator. All potential values were

corrected to the reversible hydrogen electrode (RHE) scale, and the iR-drop was fully compensated.

CFC-ICP-MS Measurements

Stability evaluations were conducted using an in-situ channel flow cell (CFC) coupled with inductively coupled plasma mass spectrometry (ICP-MS). A 10 ppb Re solution was introduced as an internal standard, and 0.1 M $HClO_4$ was employed as the electrolyte. The electrolyte flow rate from the CFC to the ICP-MS was maintained at approximately 0.4 mL min^{-1}. The working electrode potential was initially held at 1.1 V (vs. RHE) for 10 min, followed by linear sweep voltammetry (LSV) from 1.1 V to 1.65 V at a scan rate of 5 mV s^{-1} under a nitrogen atmosphere. After this LSV step, the potential was held again at 1.1 V for 10 min, and the cell was then switched to galvanostatic mode at current densities of either 100 mA mg^{-1} or 20 mA mg^{-1}, depending on the sample. Throughout both the LSV and holding steps, the total amount of dissolved Ir was quantified, and this value was compared with the corresponding generated current to calculate the S-number, providing a quantitative measure of catalyst stability under the tested conditions.

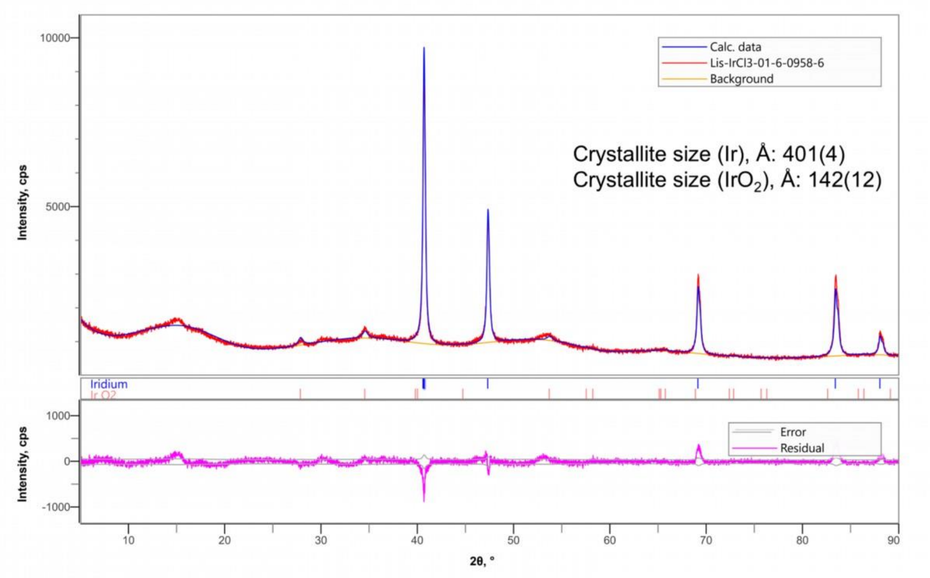

Figure S1. Rietveld refinement of XRD spectra for laser-treated $IrCl_3$: $IrCl_3$ was employed as the starting material and irradiated with a CO_2 laser at a power of 0.024 W and a scanning speed of 0.432 cm s^{-1}. The refinement results revealed crystallite sizes of ~40.1 nm for metallic Ir and ~14.2 nm for IrO_2.

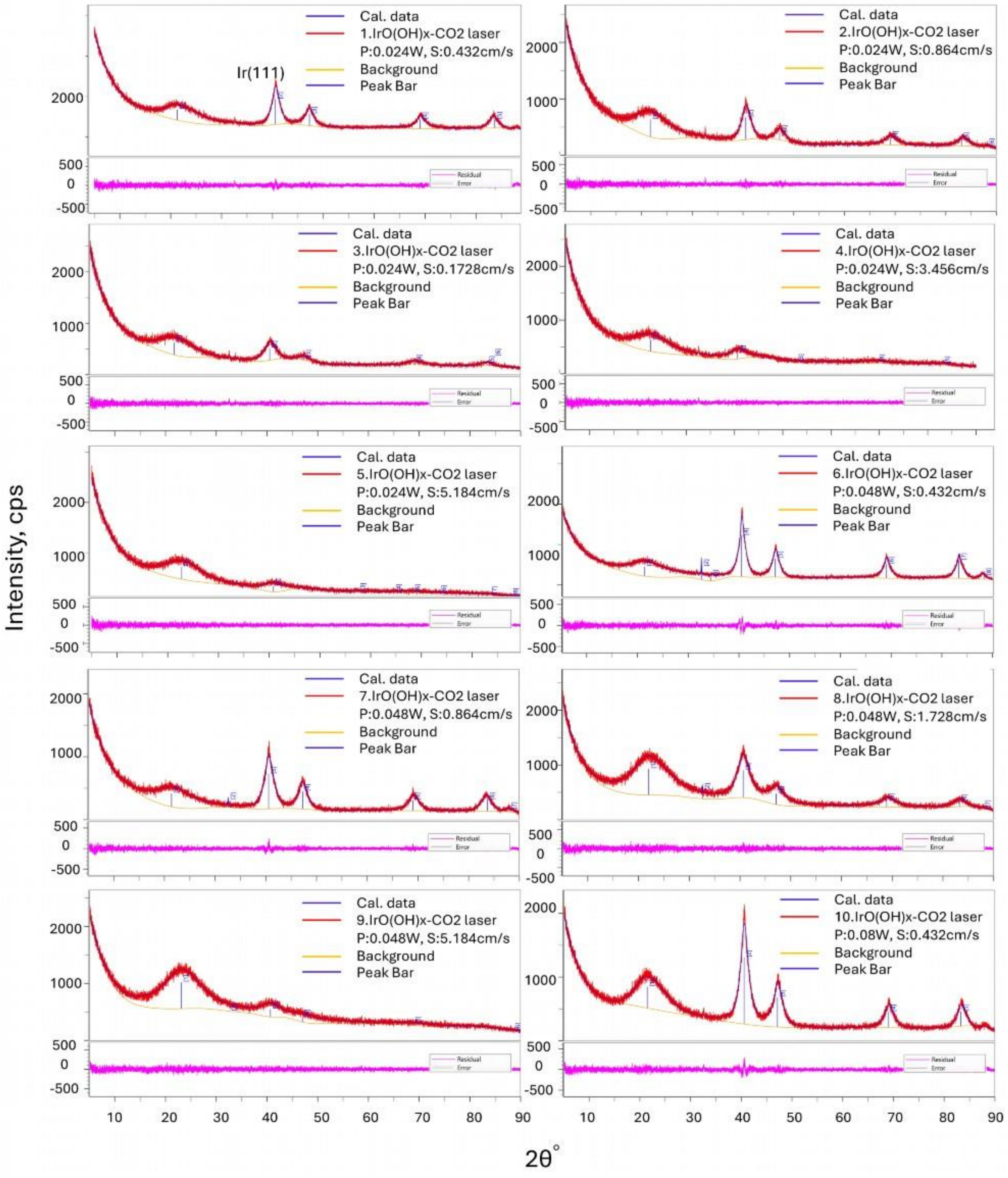

Figure S2. Rietveld refinement of XRD spectra for laser-treated $IrO(OH)_x@SiO_2$ by using CO_2 laser. $IrO(OH)_x@SiO_2$ was employed as the starting material and irradiated with a CO_2 laser at a power of 0.024, 0.048, and 0.08W, with scanning speeds of 0.432, 0.864, 0.1728, 3.456 and 5.184 cm s^{-1}.

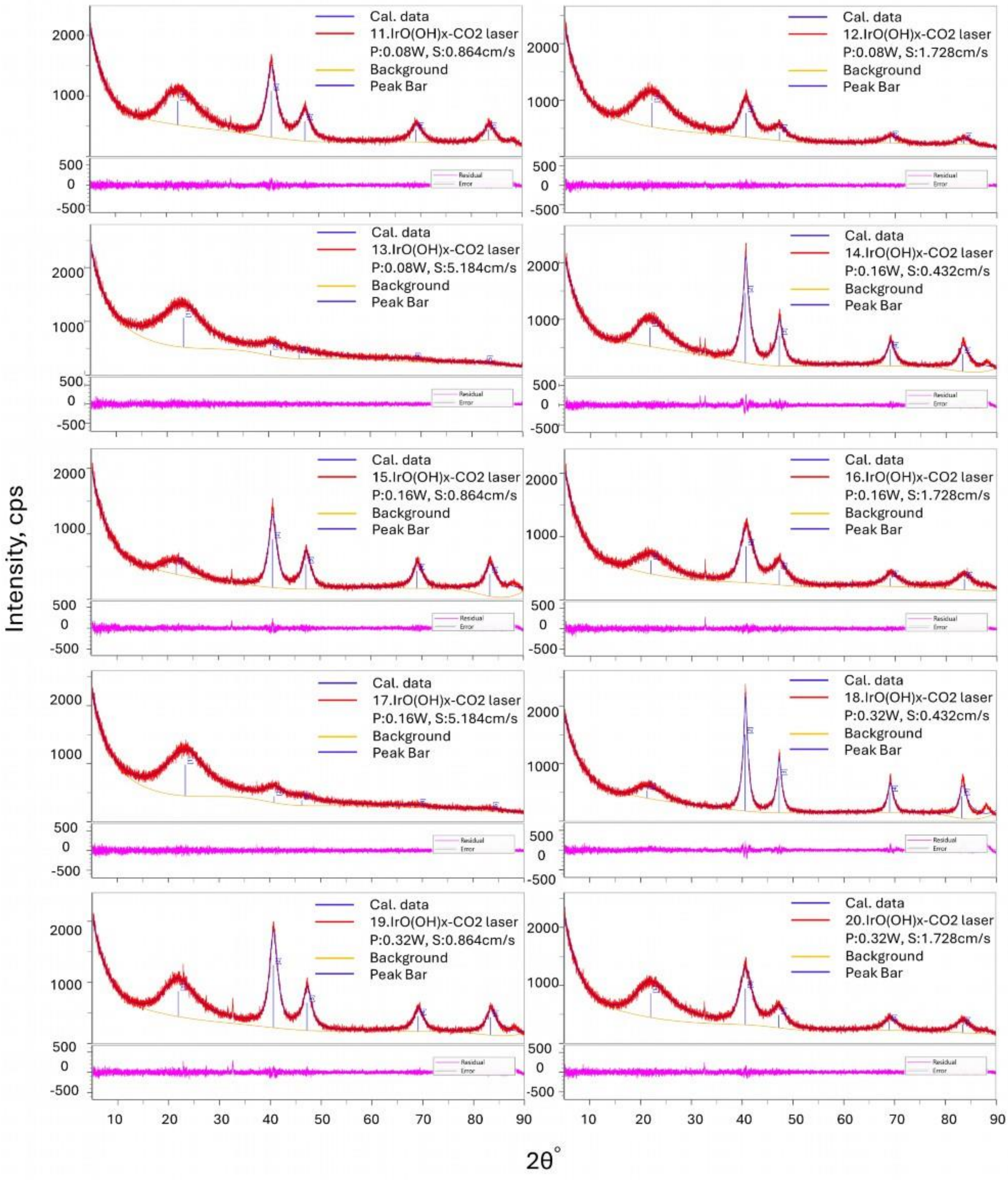

Figure S3. Rietveld refinement of XRD spectra for laser-treated $IrO(OH)_x@SiO_2$ by using CO_2 laser. $IrO(OH)_x@SiO_2$ was used as the starting material and irradiated with a CO_2 laser at power levels of 0.08, 0.16, and 0.32 W, with scanning speeds of 0.432, 0.864, 1.728, 3.456, and 5.184 cm s^{-1}.

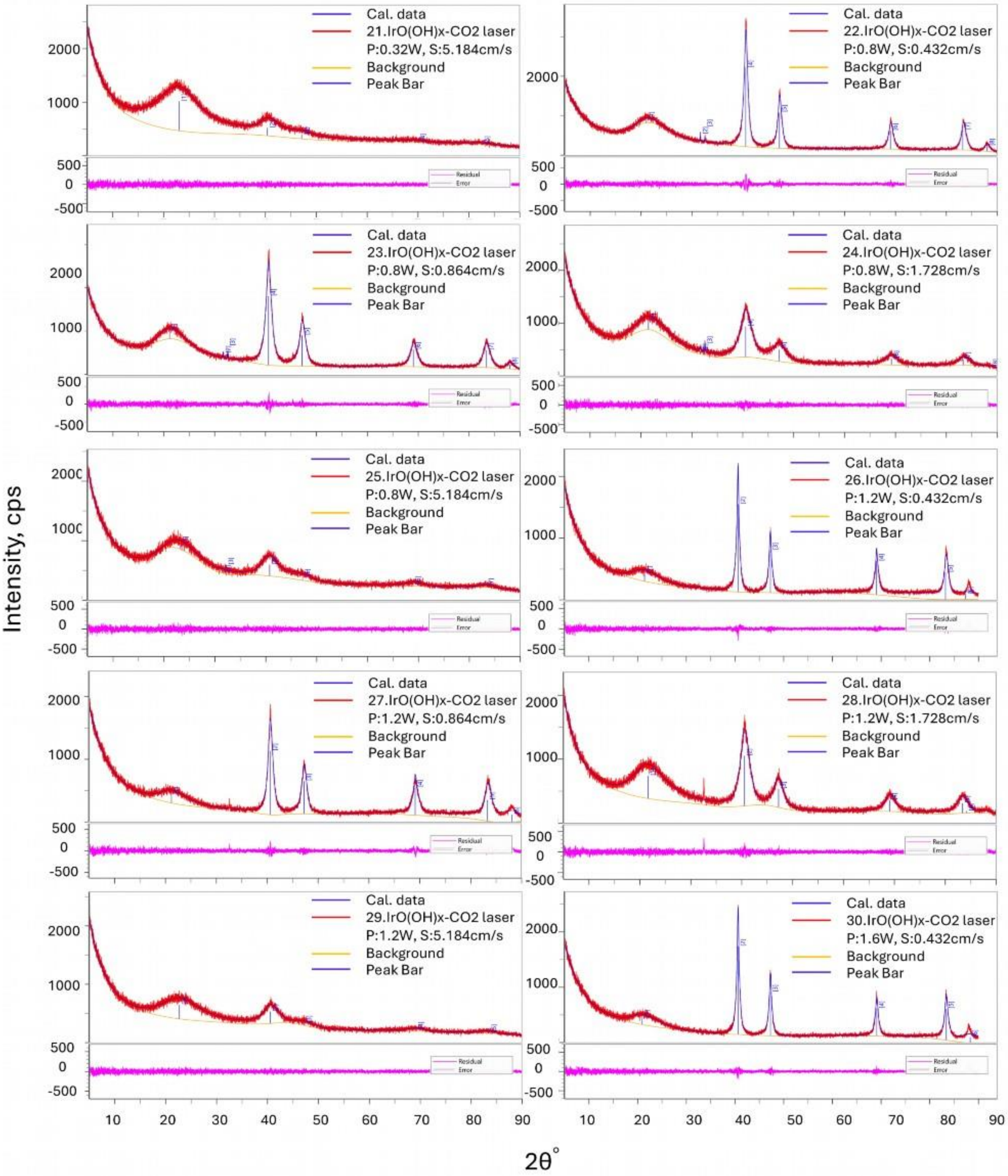

Figure S4. Rietveld refinement of XRD spectra for laser-treated $IrO(OH)_x@SiO_2$ by using CO_2 laser. $IrO(OH)_x@SiO_2$ was used as the starting material and irradiated with a CO_2 laser at power levels of 0.32, 0.8, and 1.2 W, with scanning speeds of 0.432, 0.864, 1.728, 3.456, and 5.184 cm s^{-1}.

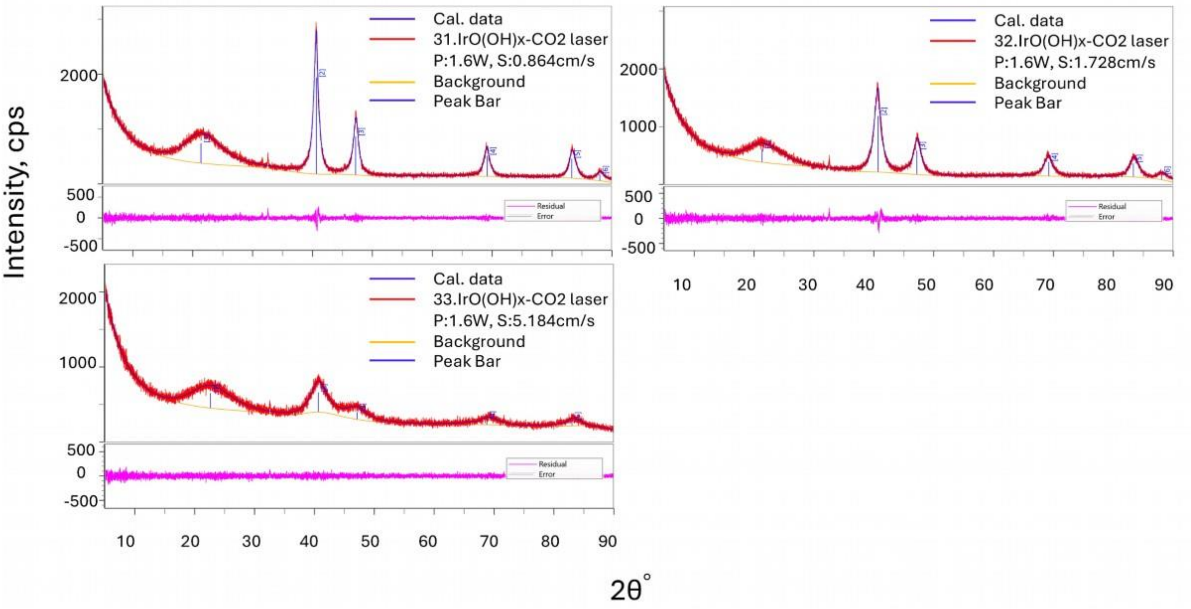

Figure S5. Rietveld refinement of XRD spectra for laser-treated $IrO(OH)_x@SiO_2$ by using CO_2 laser. $IrO(OH)_x@SiO_2$ was used as the starting material and irradiated with a CO_2 laser at power levels 1.6 W, with scanning speeds of 0.864, 1.728, and 5.184 cm s^{-1}.

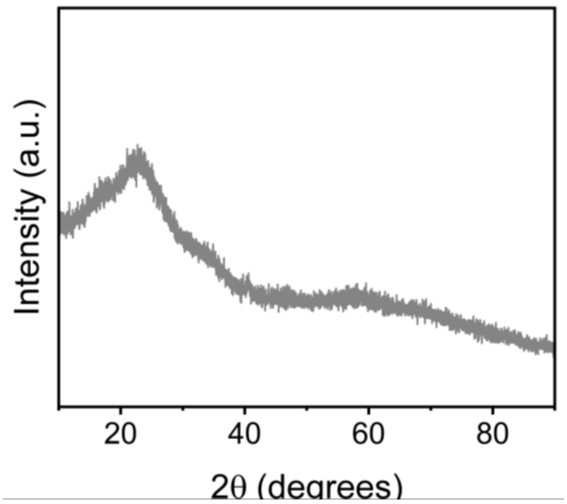

Figure S6. XRD spectra of IrO_x(300ºC)@SiO_2.

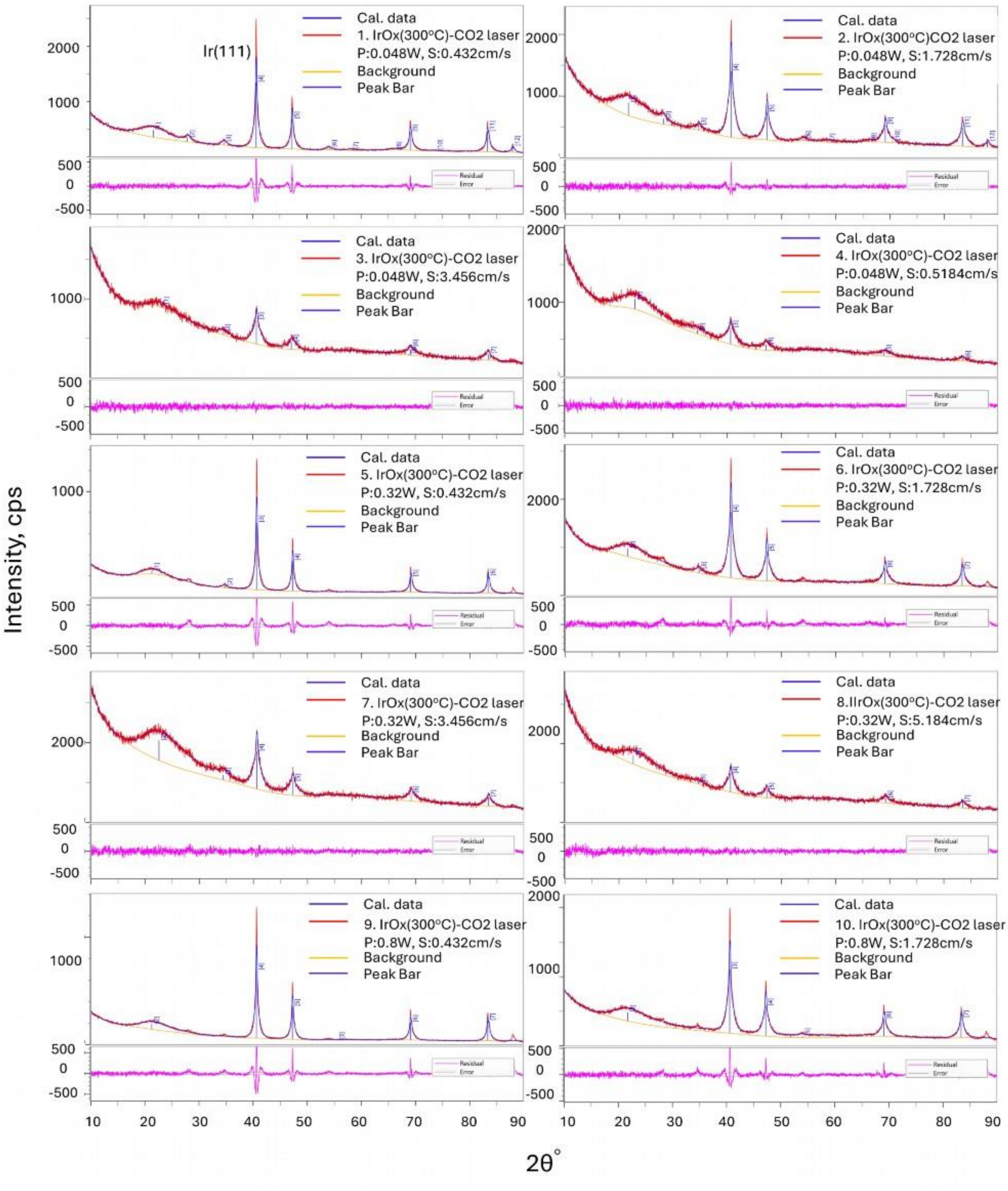

Figure S7. Rietveld refinement of XRD spectra for laser-treated IrO_x(300°C) by using CO_2 laser. IrO_x(300°C)@SiO_2 was used as the starting material and irradiated with a CO_2 laser at power levels 0.048, 0.32 and 0.8 W, with scanning speeds of 0.432, 1.728, 3.456 and 5.184 cm s^{-1}.

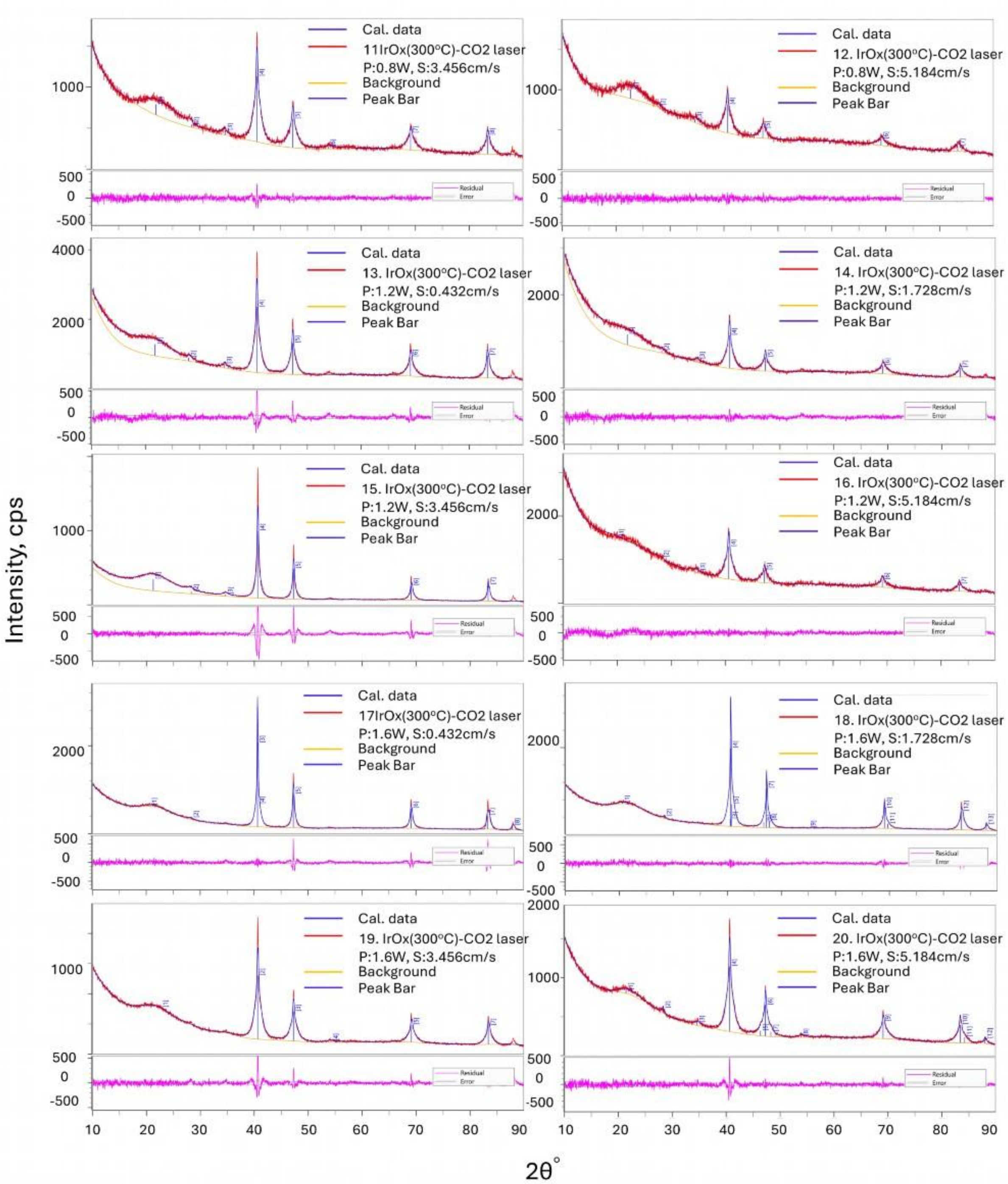

Figure S8. Rietveld refinement of XRD spectra for laser-treated IrO_x(300°C) by using CO_2 laser. IrO_x(300°C)@SiO_2 was used as the starting material and irradiated with a CO_2 laser at power levels 0.8, 1.2 and 1.6 W, with scanning speeds of 0.432, 1.728, 3.456 and 5.184 cm s^{-1}.

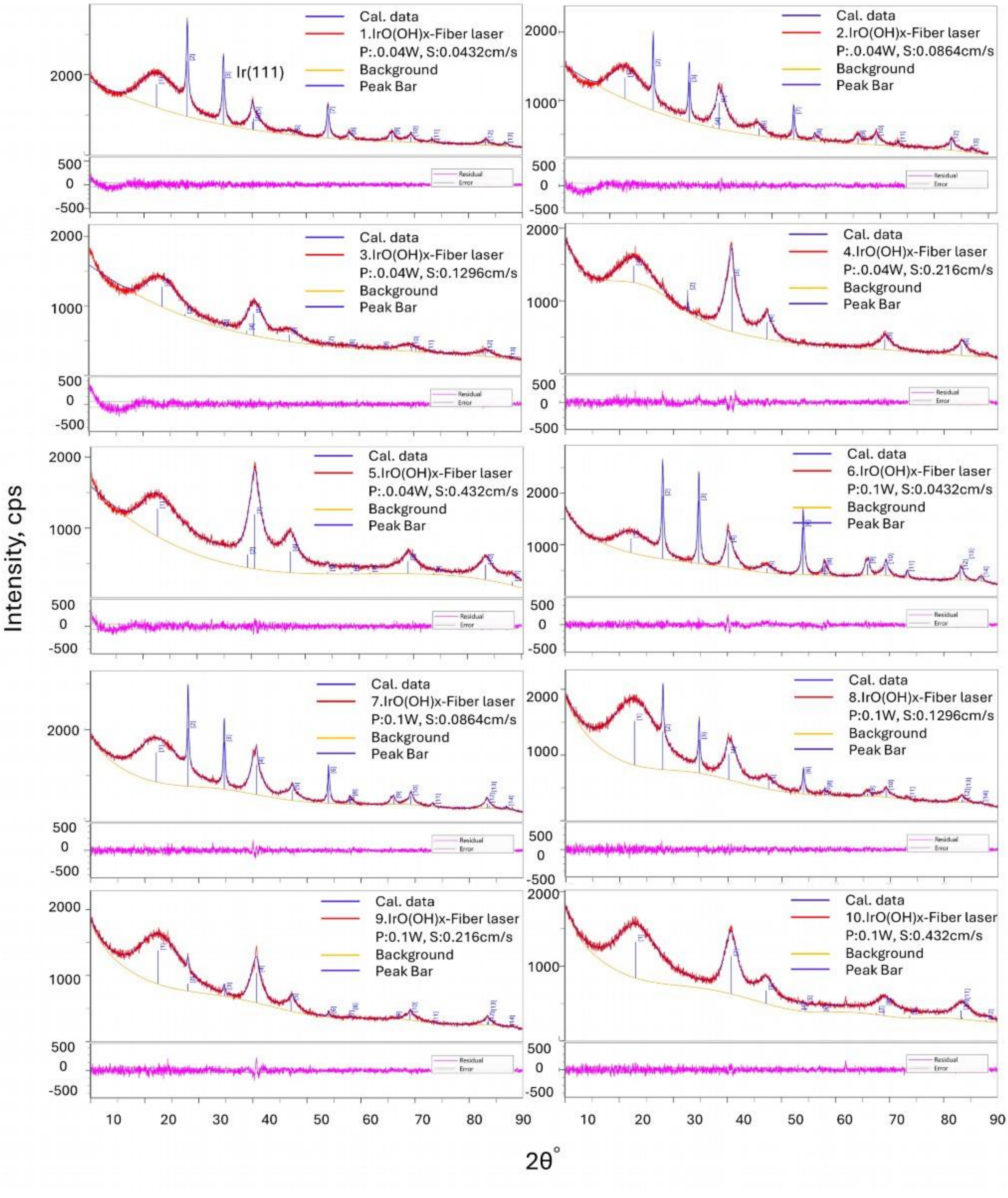

Figure S9. Rietveld refinement of XRD spectra for laser-treated IrO(OH)$_x$ by using fiber laser. IrO(OH)$_x$@SiO$_2$ was used as the starting material and irradiated with a fiber laser at power levels 0.04, and 0.1 W, with scanning speeds of 0.0432, 0.0864, 0.1296 and 0.432 cm s^{-1}.

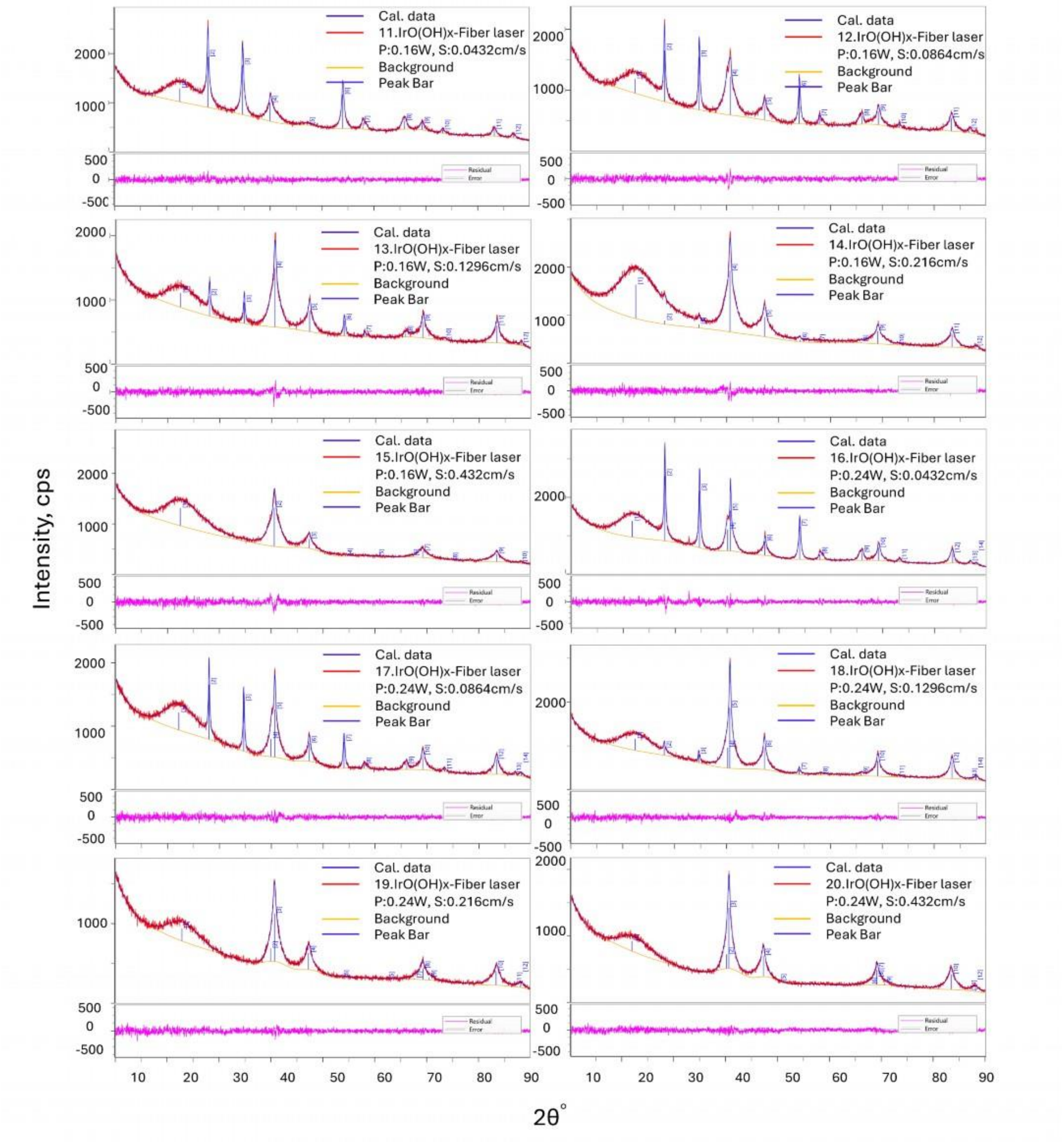

Figure S10. Rietveld refinement of XRD spectra for laser-treated $IrO(OH)_x$ by using fiber laser. $IrO(OH)_x$@SiO_2 was used as the starting material and irradiated with a fiber laser at power levels 0.04, and 0.1 W, with scanning speeds of 0.0432, 0.0864, 0.1296, 0.216 and 0.432 cm s^{-1}.

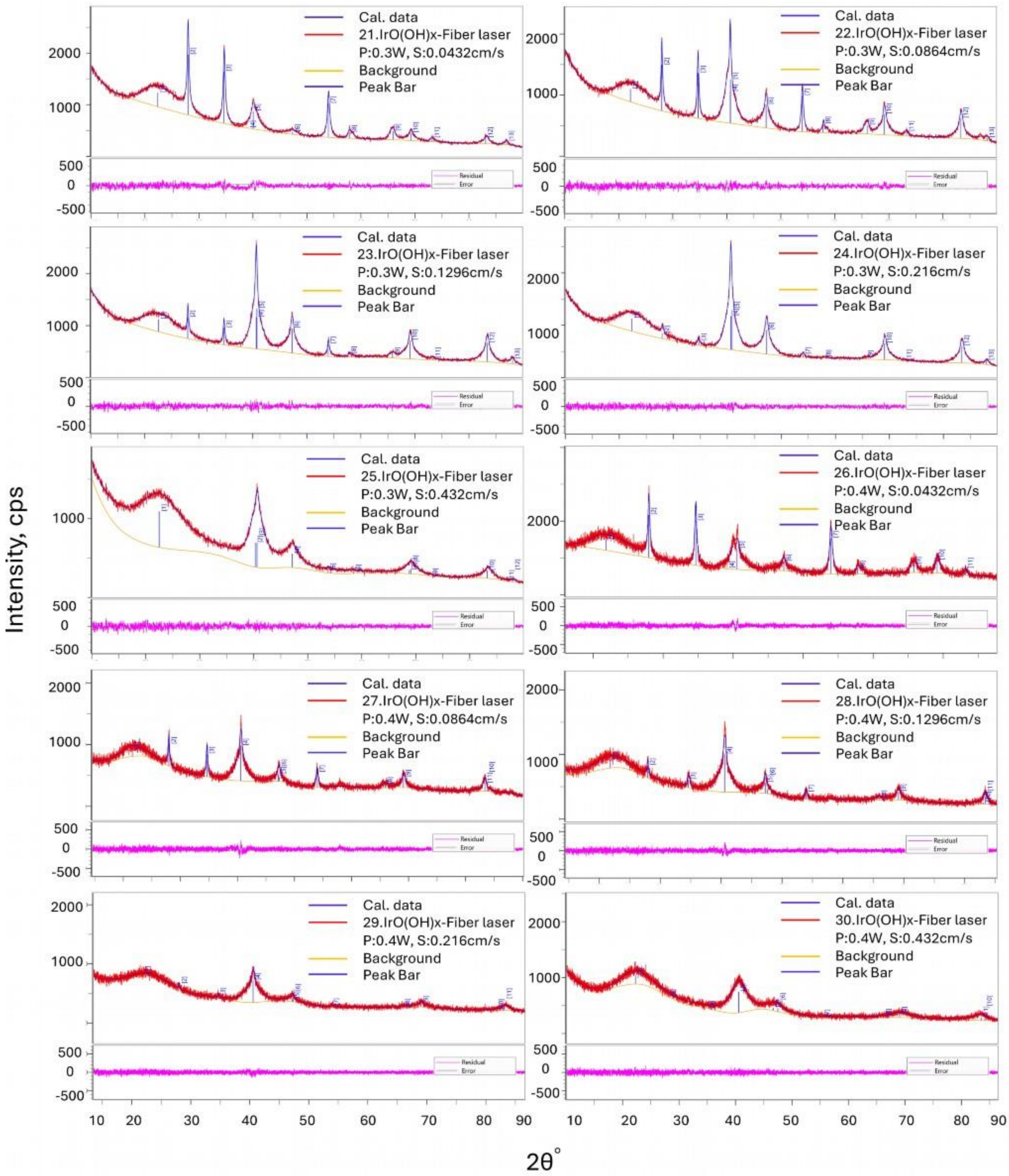

Figure S11. Rietveld refinement of XRD spectra for laser-treated $IrO(OH)_x$ by using fiber laser. $IrO(OH)_x@SiO_2$ was used as the starting material and irradiated with a fiber laser at power levels 0.3, and 0.4 W, with scanning speeds of 0.0432, 0.0864, 0.1296, 0.216 and 0.432 cm s^{-1}.

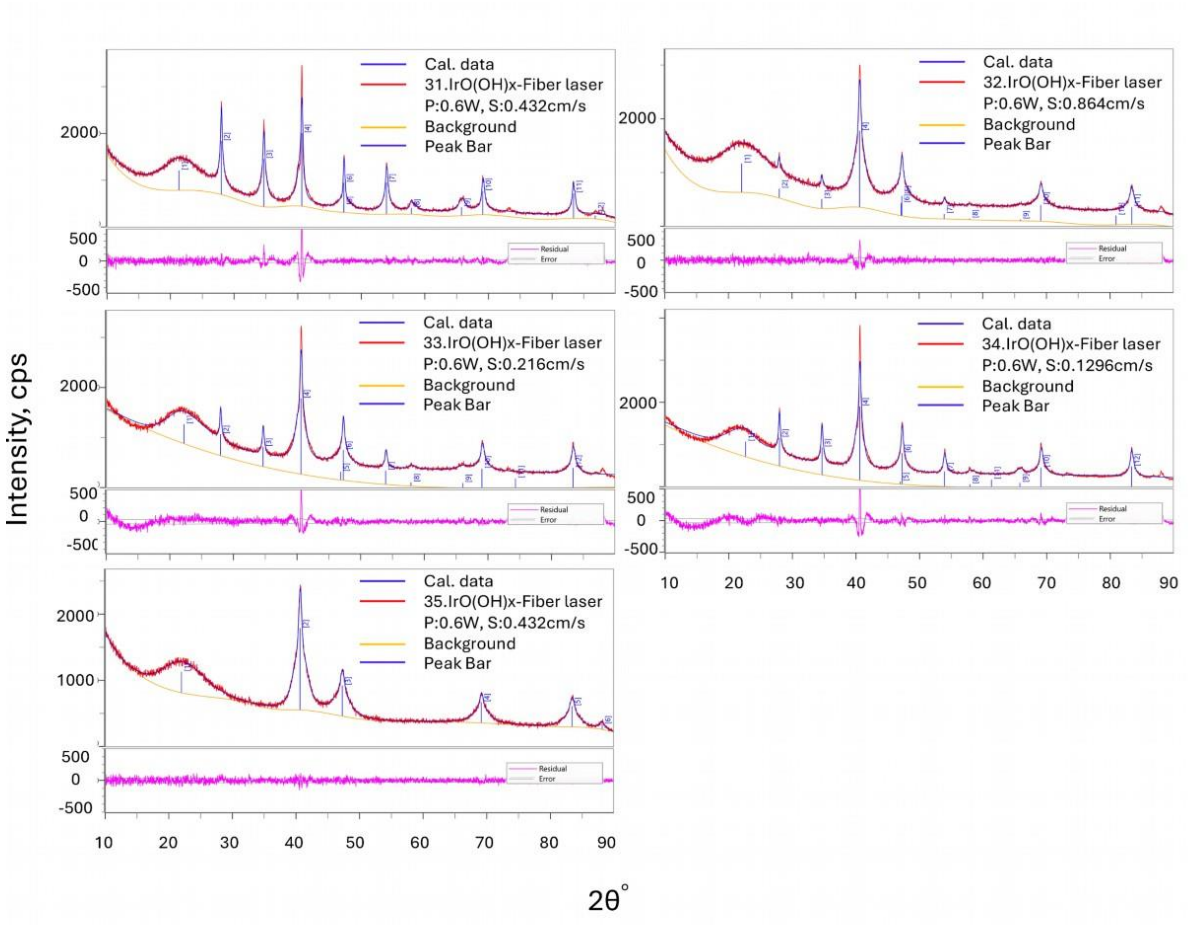

Figure S12. Rietveld refinement of XRD spectra for laser-treated $IrO(OH)_x$ by using fiber laser. $IrO(OH)_x@SiO_2$ was used as the starting material and irradiated with a fiber laser at power levels 0.6 W, with scanning speeds of 0.0432, 0.0864, 0.1296, 0.216 and 0.432 cm s^{-1}.

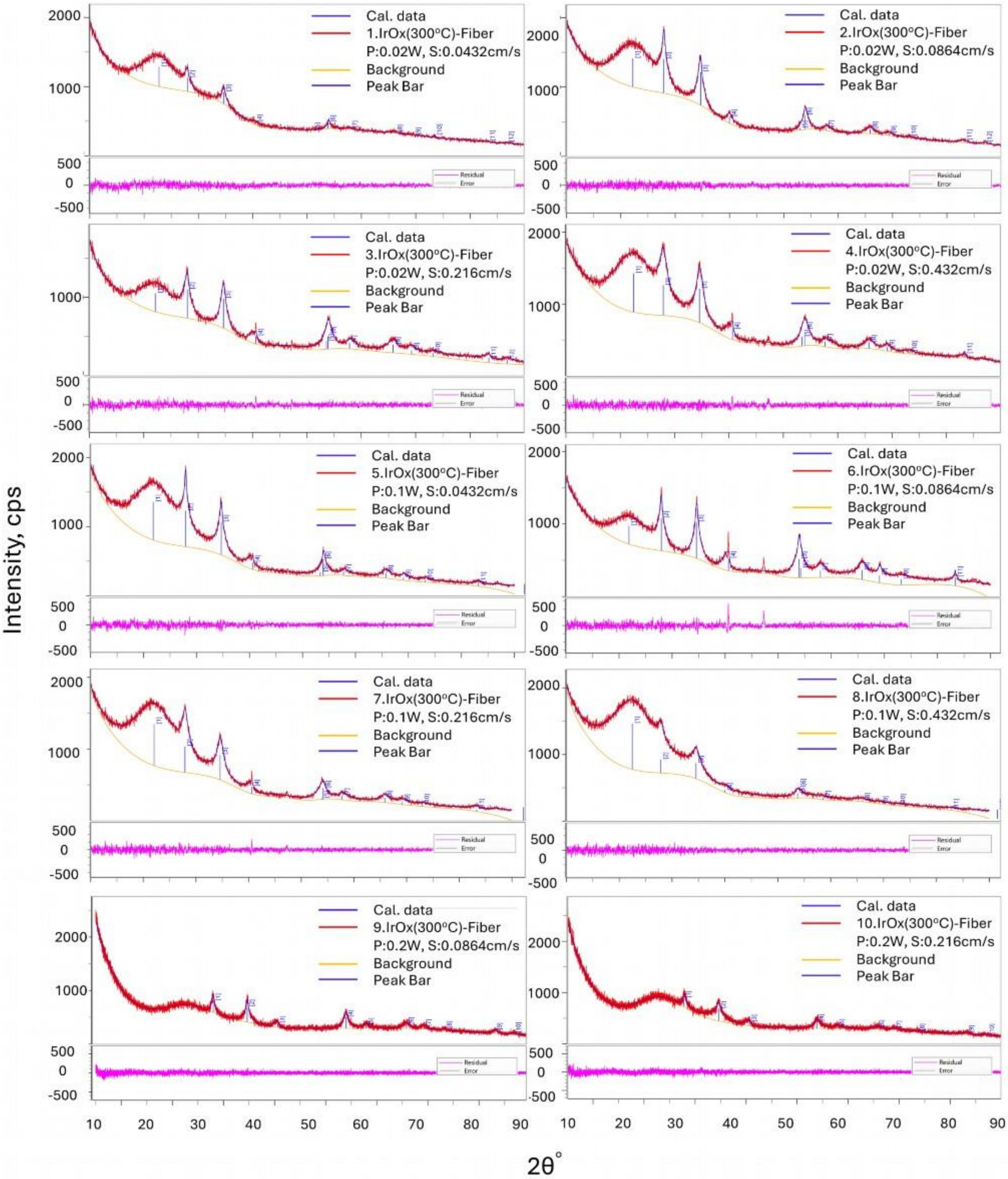

Figure S13. Rietveld refinement of XRD spectra for laser-treated IrO_x(300°C) by using fiber laser. IrO_x(300 °C)@SiO_2 was used as the starting material and irradiated with a fiber laser at power levels 0.02,0.1 and 0.2 W, with scanning speeds of 0.0432, 0.0864, 0.216 and 0.432 cm s^{-1}.

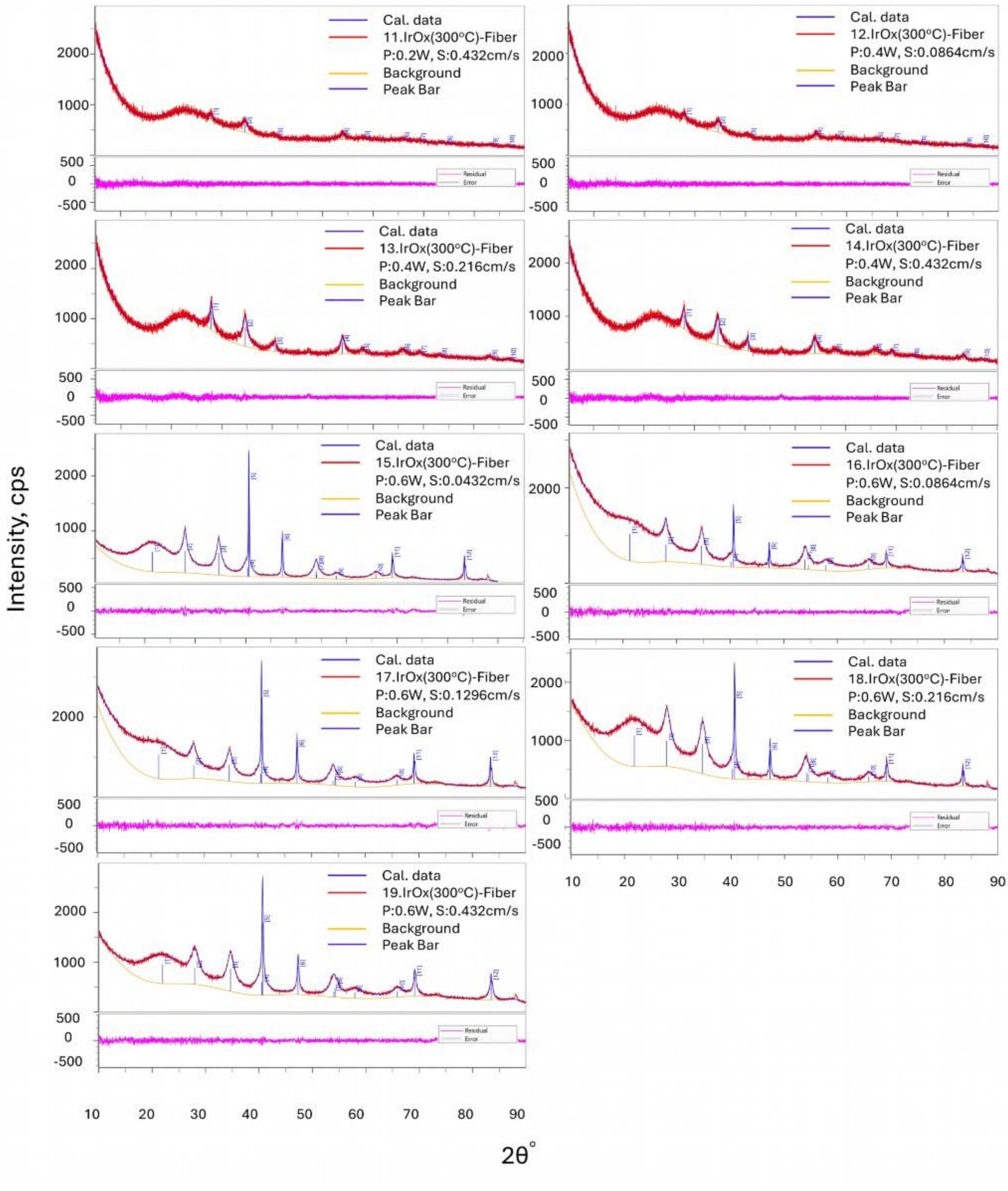

Figure S14. Rietveld refinement of XRD spectra for laser-treated IrO_x(300ºC) by using fiber laser. IrO_x(300 ºC)@SiO_2 was used as the starting material and irradiated with a fiber laser at power levels 0.2, 0.4 and 0.6 W, with scanning speeds of 0.0432, 0.0864, 0.216 and 0.432 cm s^{-1}.

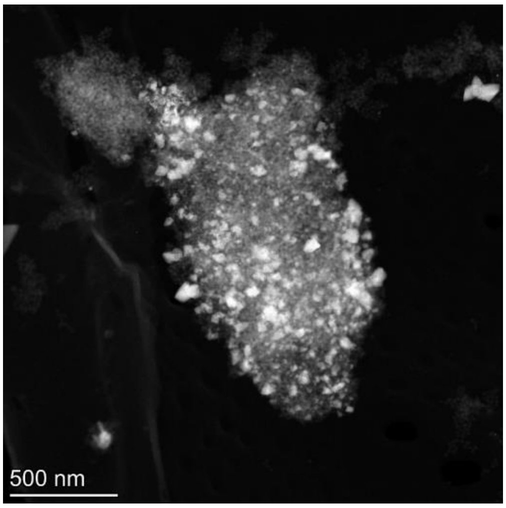

Figure S15. HAADF-STEM image of laser-treated $IrO(OH)_x@SiO_2$ using a fiber laser. Irradiation at 0.1 W with a scanning speed of 0.0432 cm s^{-1} results in the formation of large crystallites, accompanied by the agglomeration of adjacent nanoparticles due to the damage of the SiO_2 shell.

Note S1

As shown in Figure S15, the gas signals for $IrO(OH)_x@SiO_2$ are significantly higher than those for blank SiO_2. Notably, when $IrO(OH)_x@SiO_2$ is treated with a CO_2 laser, the H_2 signal increases by two orders of magnitude (from 10^{-10} to 10^{-8}), whereas for blank SiO_2, the H_2 signal shows only a slight increase.

Furthermore, XRD results from Figure S16 indicate that the SiO_2 blank sample remains structurally unchanged under laser irradiation, suggesting that the observed changes in CO_2, CO, and other gas signals originate from oxygen atoms released during the decomposition of absorbed water.

In contrast, the $IrO(OH)_x@SiO_2$ sample exhibits a significantly stronger CO_2^+ (m/z = 44) and CO/N_2^+ (m/z = 28) signal, likely due to the oxidation of organic residues from the microemulsion used during synthesis. The CO/N_2^+ (m/z = 28) and O^+ (m/z = 16) signals serve as fragment peaks, further confirming their presence. Additionally, Figure S15a shows fluctuations in the O_2^+ (m/z = 32) signal, which are attributed to the rapid release of other gas species.

Figure 16 presents the XRD data of the solid-phase components remaining after *operando* mass spectrometry (MS) analysis. Notably, the XRD spectrum of IrOx(300 °C)@SiO_2, treated with a fiber laser in an argon environment, reveals the presence of crystalline Ir and iridium oxide. However, in an air environment, only iridium oxide is observed (Figure 4b). Since all *operando* MS experiments were conducted in argon, the low oxygen availability was insufficient for the formation of iridium oxide. Nevertheless, the small amount of iridium oxide detected in the solid-phase product suggests that laser irradiation can induce the transformation of amorphous iridium oxide into a crystalline phase, even in an inert gas environment. For other reactions, the formation of crystalline iridium was observed, consistent with reactions performed in air (Figure 2).

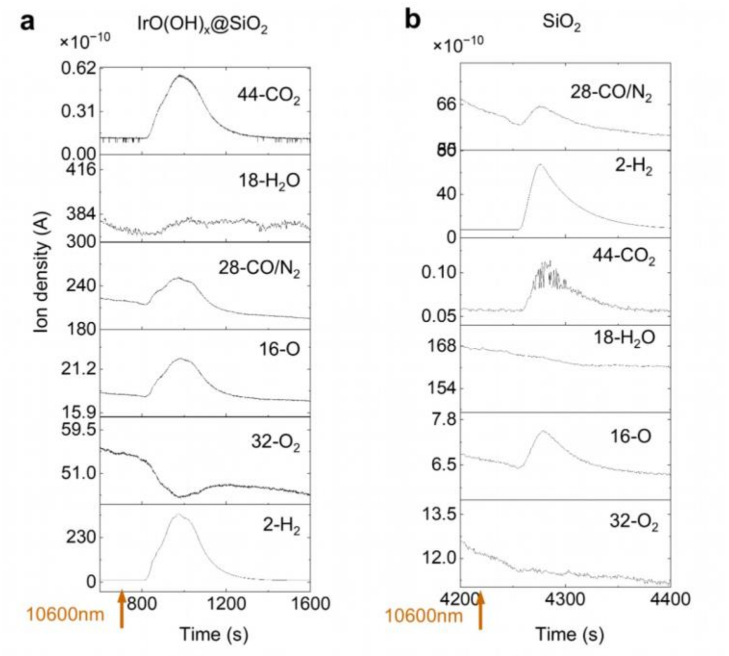

Figure S16. MS spectra for H_2O^+ (m/z = 18), CO_2^+ (m/z = 44), NO^+ (m/z = 30), CO/N_2^+ (m/z = 28), O^+ (m/z = 16), O_2^+ (m/z = 32), and H_2^+ (m/z = 2)are shown for: (**a**) $IrO(OH)_x@SiO_2$ as the starting material, irradiated with a CO_2 laser at 0.8 W and a scanning speed of 0.216 cm s^{-1}. (**b**) SiO_2 as the starting material, irradiated with a CO_2 laser at 0.8 W and a scanning speed of 0.216 cm s^{-1}.

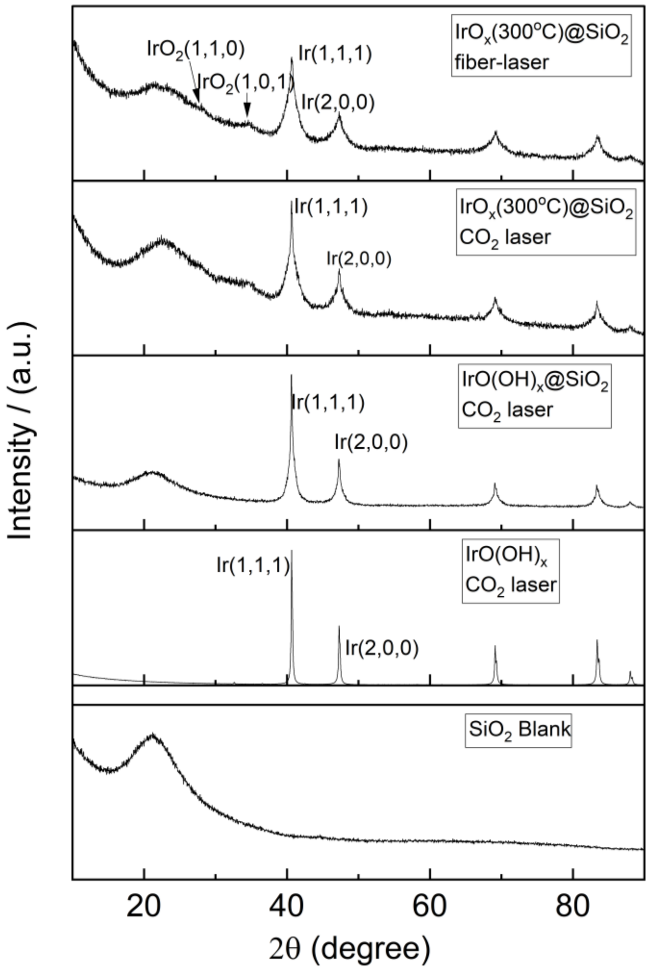

Figure S17. XRD spectra of materials after *operando* MS measurements. All samples were irradiated with a laser under an argon atmosphere.

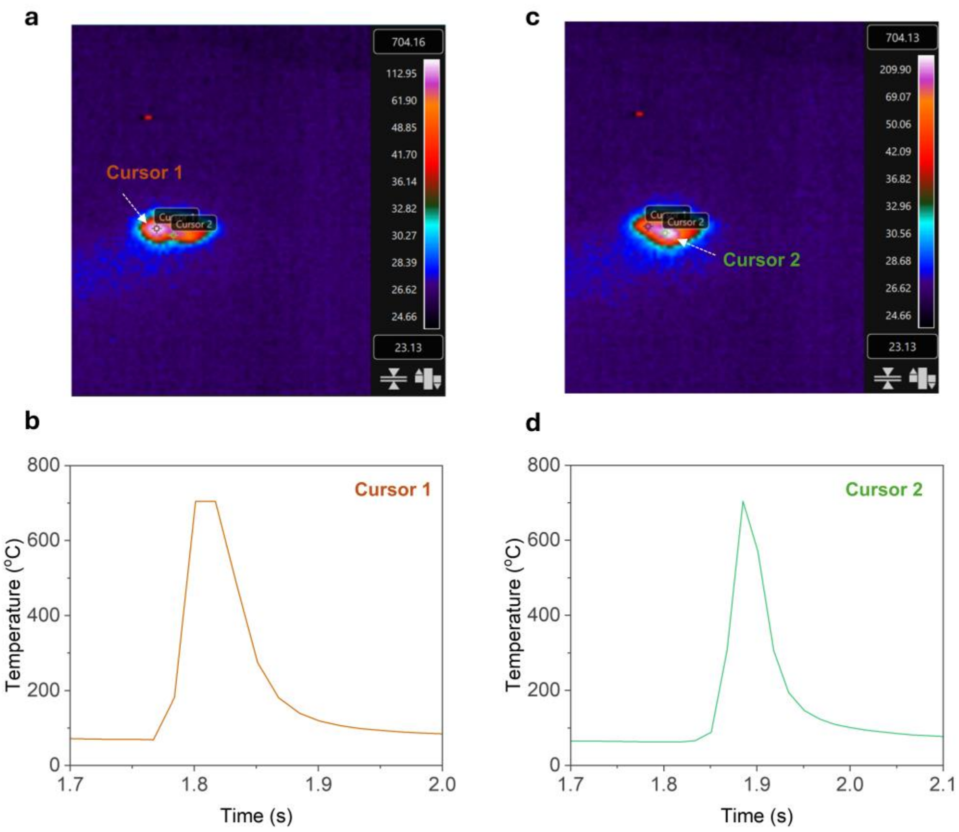

Figure S18: Real-time thermal response during CO_2 laser irradiation using $IrO(OH)_x@SiO_2$ as the precursor, under a laser power density of 32 W cm^{-2} and a scanning speed of 5.2 cm s^{-1}. (a) Infrared image of the sample at cursor 1. (b) Corresponding maximum temperature profile at cursor 1 as the laser beam passes. (c) Infrared image of the sample at cursor 2. (d) Corresponding maximum temperature profile at cursor 2 as the laser beam passes.

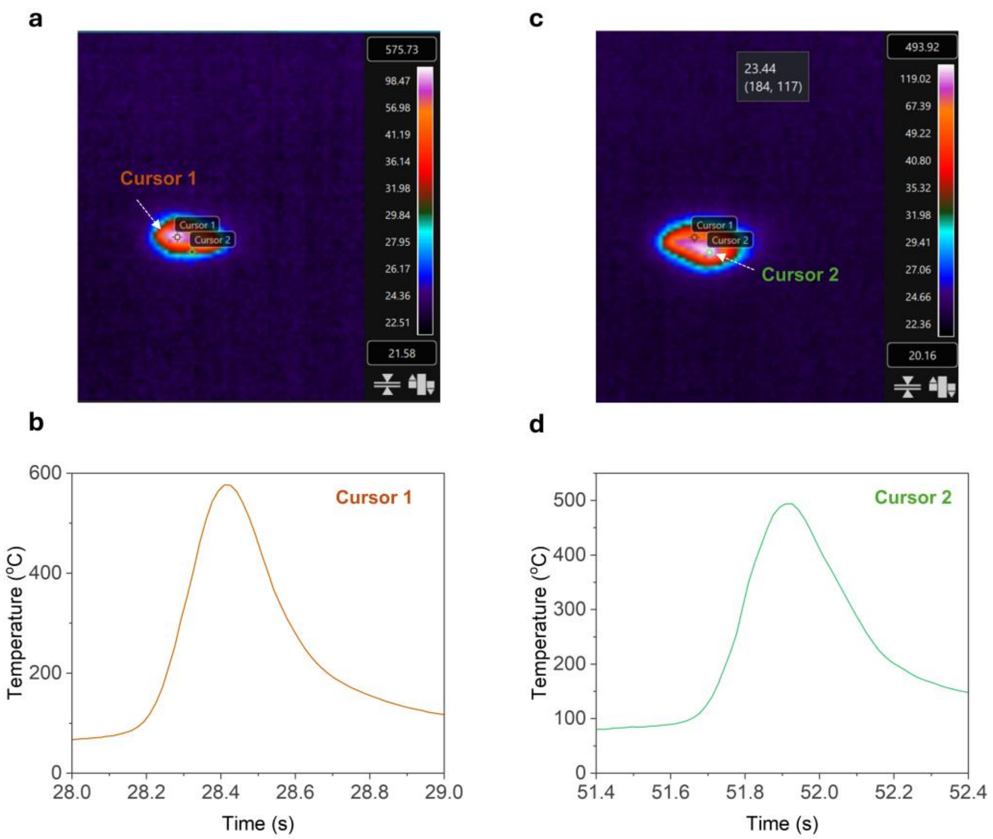

Figure S19. Real-time thermal response during fiber laser irradiation using IrO_x(300 °C)@SiO_2 as the precursor, under a laser power density of 1.67 kW cm^{-2} and a scanning speed of 0.432 cm s^{-1}. (a) Infrared image of the sample at cursor 1. (b) Corresponding maximum temperature profile at cursor 1 as the laser beam passes. (c) Infrared image of the sample at cursor 2. (d) Corresponding maximum temperature profile at cursor 2 as the laser beam passes.

Note: as shown in Figure S18-19, during the CO_2 laser synthesis process, the temperature at two selected positions rapidly reached the upper detection limit of the infrared (IR) camera (704 °C) when the laser beam passed through. It is reasonable to infer that the actual temperature exceeded this limit, which could induce rapid thermal decomposition of $IrO(OH)_x$ and facilitate the formation of metallic Ir. In comparison, the thermal effect induced by the fiber laser was more moderate. At the selected position 1, the maximum recorded temperature was 575 °C, which is sufficient to promote the transformation from the amorphous to the crystalline phase.

It should be noted that the temporal and temperature resolution of the IR camera imposes limitations on the accuracy of the measured thermal response. A more precise evaluation of the temperature evolution during laser irradiation would require advanced thermal diagnostic tools with higher sensitivity and faster response time.

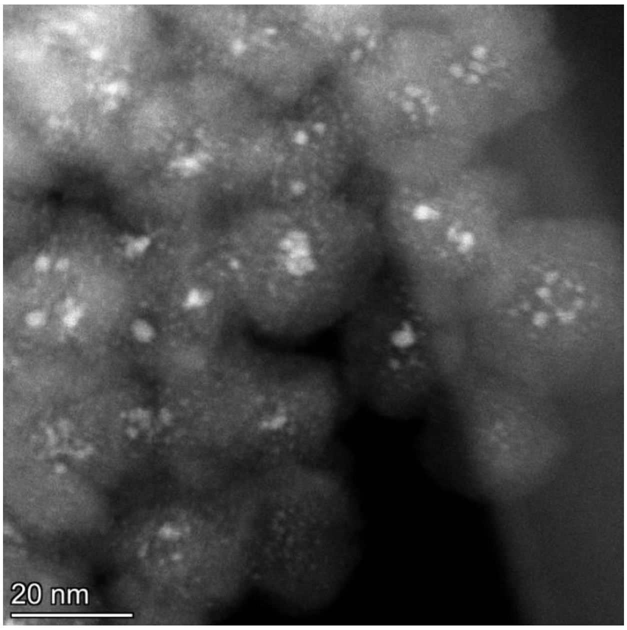

Figure S20. HAADF-STEM image of $IrO(OH)_x$@SiO_2.

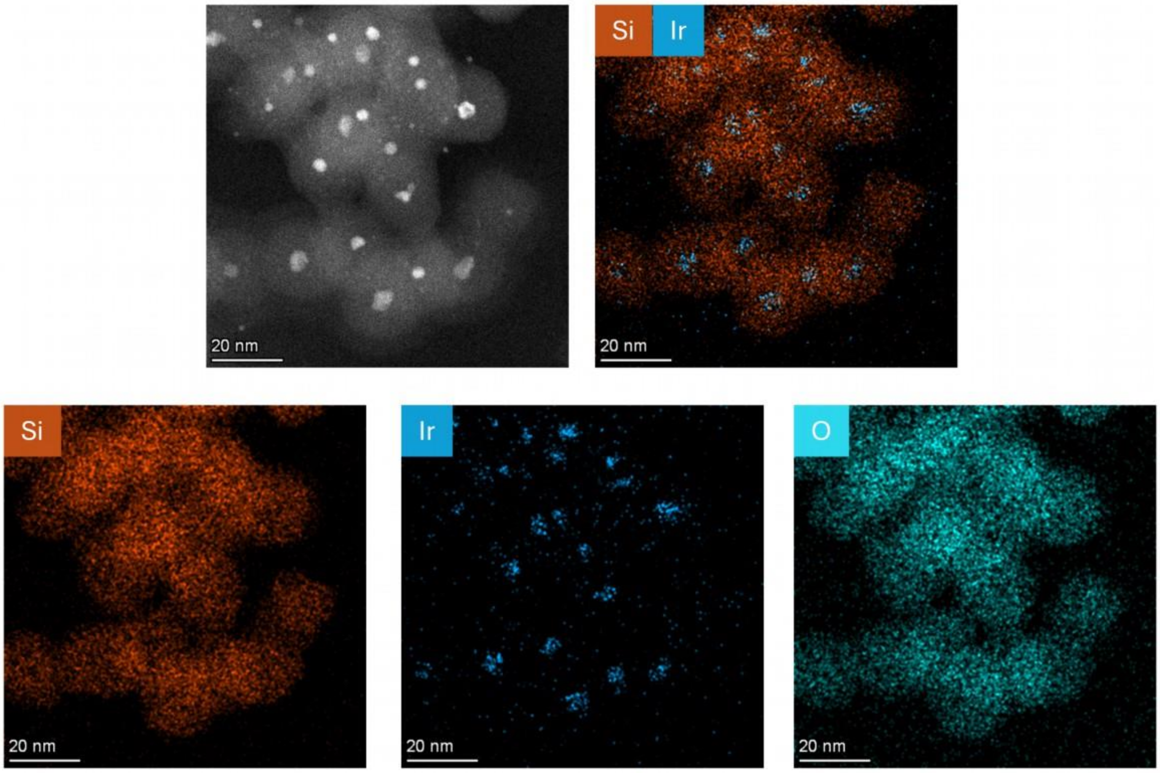

Figure S21. HAADF-STEM images and elemental maps of Lis-IrO_2 in the silica shell. Red represents silicon, blue represents iridium, and cyan represents oxygen. The images confirm that all iridium oxide nanoparticles are uniformly encapsulated within the SiO_2 shell and exhibit consistent size distribution.

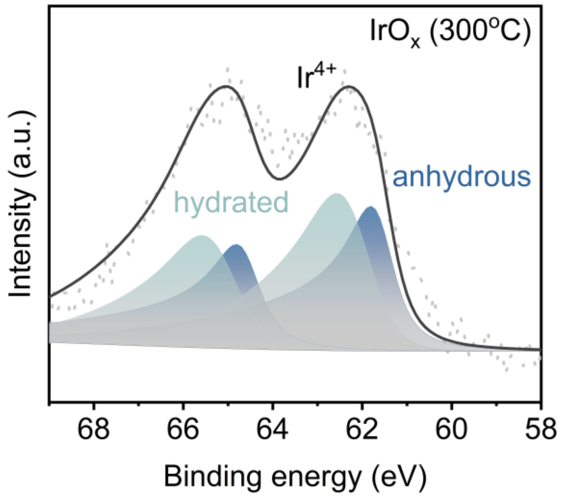

Figure S22. XPS spectra of Ir 4f of IrO_x(300 ºC).

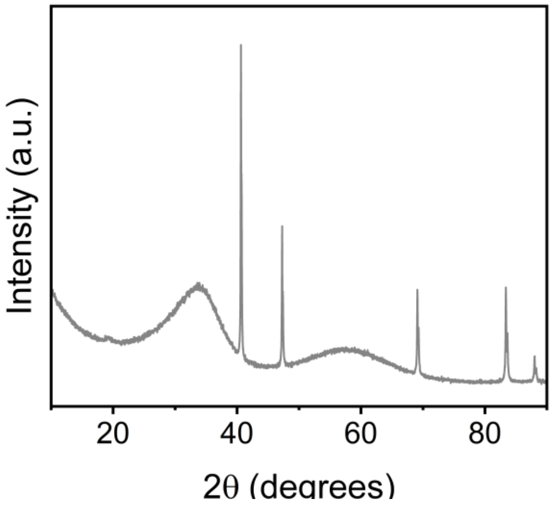

Figure S23. XRD spectra of Alfa Aesar IrO_2.

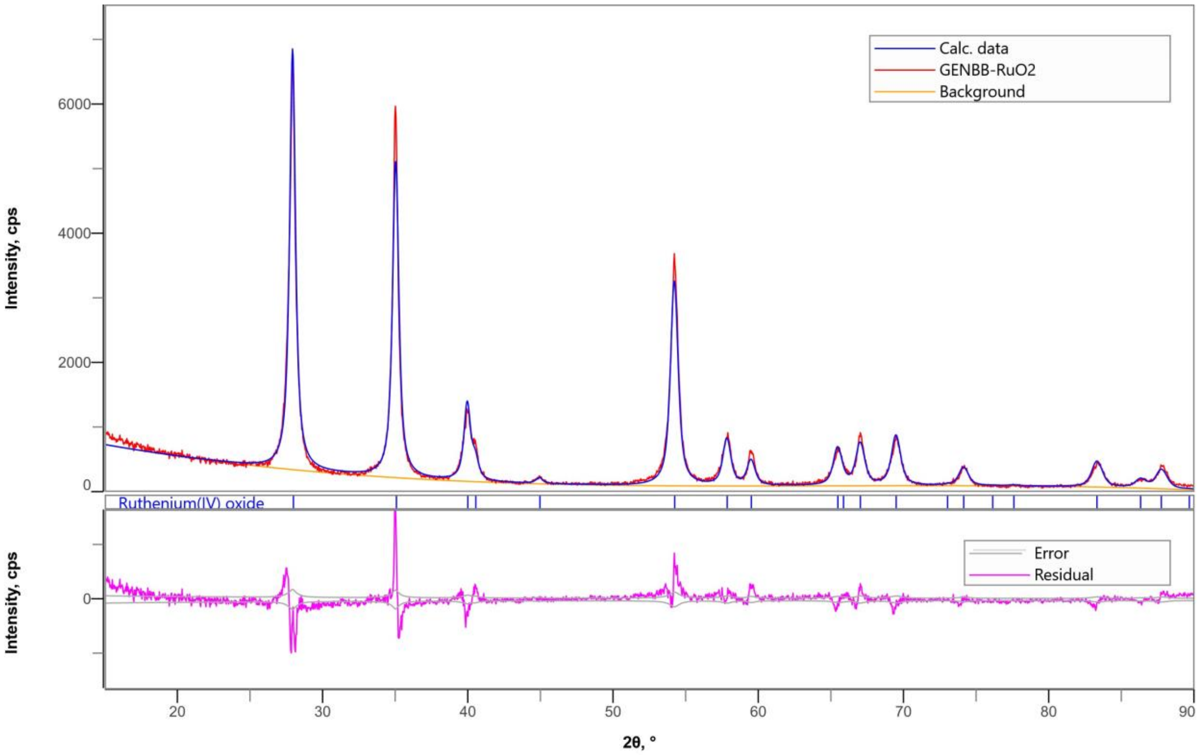

Figure S24 XRD spectra of crystalline RuO_2: The annealed RuO_2 shows a crystalline size of 19 nm obtained by WPPF analysis.

Note S2

RDE measurement

Before the start of the measurement protocol, the impedance is measured using electrical impedance spectroscopy (EIS). 80 % of the impedance is compensated during the measurement. The remaining 20 % is compensated, post-process during data analysis. During all measurements, the electrolyte is saturated by bubbling nitrogen gas. At the beginning, the working electrode is kept in galvanostatic mode at 0 A current (open circuit potential (OCP)) for 10 min to ensure the full saturation of the electrolyte with N_2. For catalyst activation, the working electrode is then cycled for 200 CVs between 0.05 V vs. RHE and 1.39 V vs. RHE with a scan rate of 200 mV/s. For catalyst characterization, 3 CVs in the same potential range but with a scan rate of 50 mV/s are recorded. Lastly, four linear sweep voltammetry (LSV) measurements are conducted from 1.29 V vs. RHE to 1.64 V vs. RHE with a scan rate of 10 mV/s. For analysis, the second CV and the first LSV were used.

In the data analysis, the raw data is converted to potential vs. RHE, the remaining IR-drop is compensated, and the measured currents are normalized by the geometric surface area of the RDE tip and by the iridium loading of the catalyst.

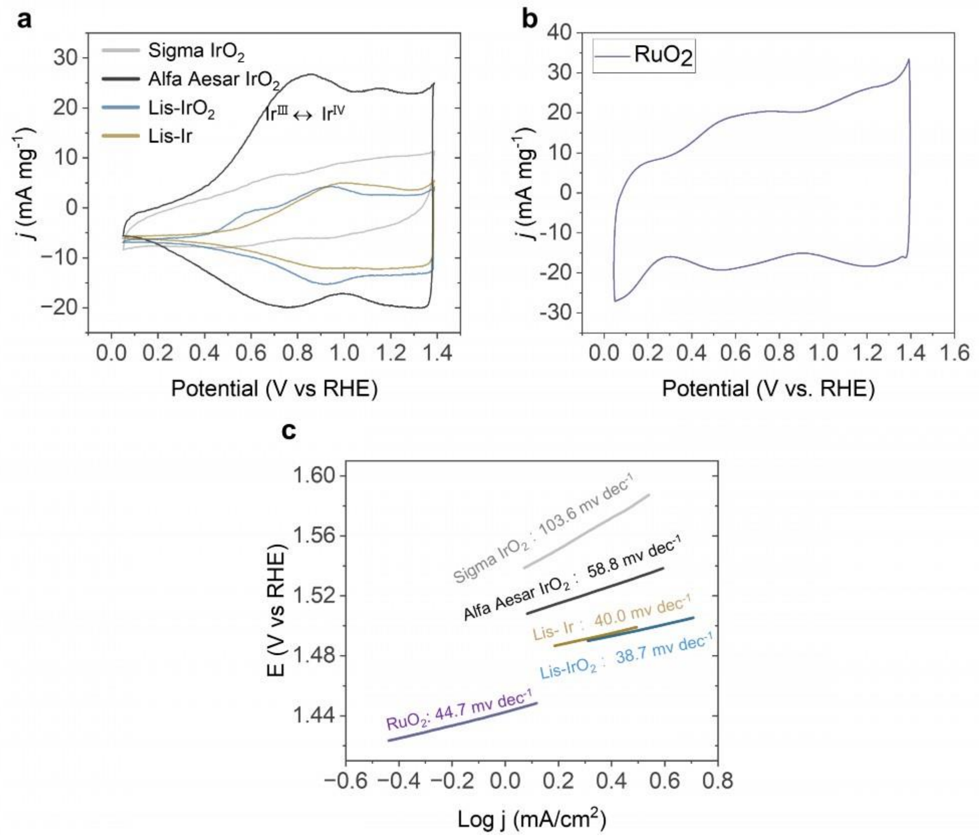

Figure S25. RDE measurements of laser-induced synthesized catalyst, reference IrO_2 and RuO_2 catalyst. a. Mass normalized cyclic voltammetry of IrO_2 catalysts measured with a scan rate of 50 mV s^{-1} between 0.05 – 1.39 V_{RHE}. b. Mass normalized cyclic voltammetry of IrO_2 catalysts measured with a scan rate of 50 mV s^{-1} between 0.05 – 1.39 V_{RHE}.**c.** Tafel slopes of studied catalysts.

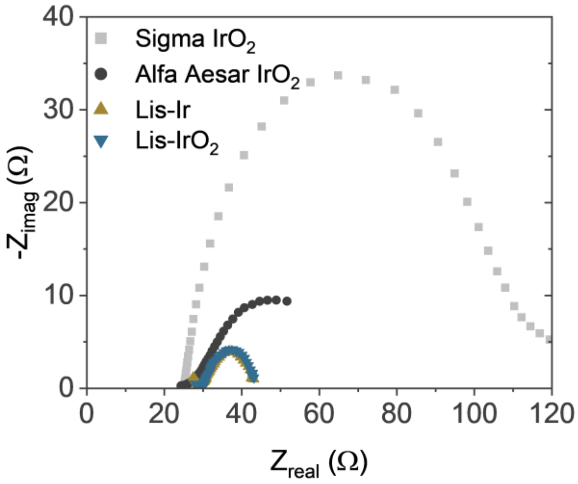

Figure S 26. Electrochemical impedance spectroscopy (EIS) spectra of the reference IrO_2 catalysts, Lis-Ir, and Lis-IrO_2, measured at 1.6 V vs. RHE over a frequency range from 100 kHz to 1 Hz.

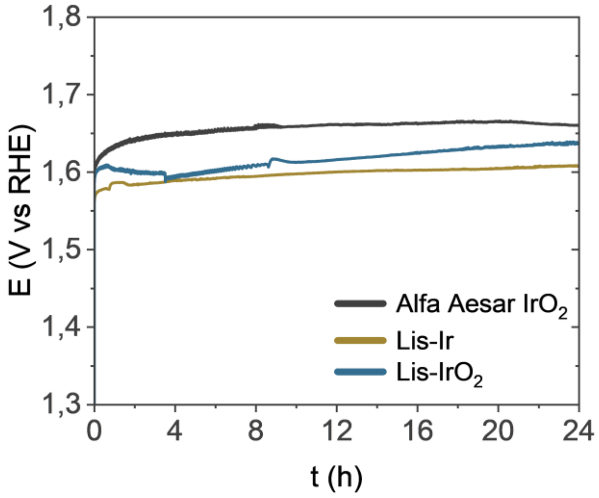

Figure S27, Chronopotentiometric stability test of Alfa Aesar IrO_2, Lis-Ir and Lis-IrO_2 at a constant current density of 10 mA cm^{-2} over 24 h.

Note: in the chronoamperometric (CA) measurements, a significant decline in activity was observed for the commercial Alfa Aesar IrO_2 after 24 hours of operation. In contrast, both Lis-Ir and Lis-IrO_2 exhibited enhanced stability under identical conditions. Notably, the Lis-IrO_2 sample was strongly affected by the accumulation of microscopic oxygen bubbles during the test, as evidenced by the marked potential fluctuations in the early stage of the measurement. This phenomenon has also been reported in previous studies.[2,3]

To gain deeper insight into the degradation mechanism of iridium-based catalysts, we conducted on-line ICP-MS measurements using a channel flow cell (CFC) configuration, enabling real-time monitoring of Ir dissolution during OER.

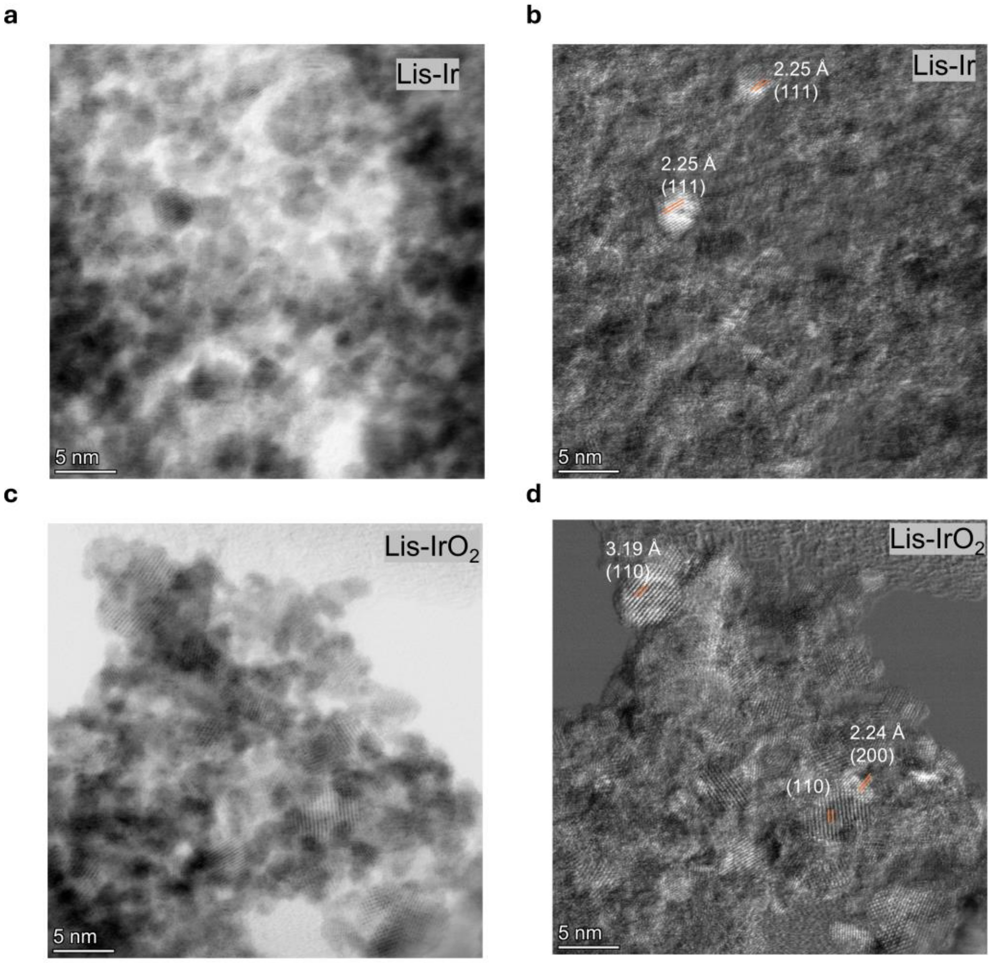

Figure S28. TEM analysis of the laser-induced synthesized catalysts after long-term stability testing. (a) STEM inner annular dark-field (DF-I) image of Lis-Ir after durability testing. (b) Corresponding bright-field (BF) image of Lis-Ir. (c) STEM inner annular dark-field (DF-I) image of Lis-IrO_2 after durability testing. (d) Corresponding bright-field (BF) image of Lis-IrO_2.

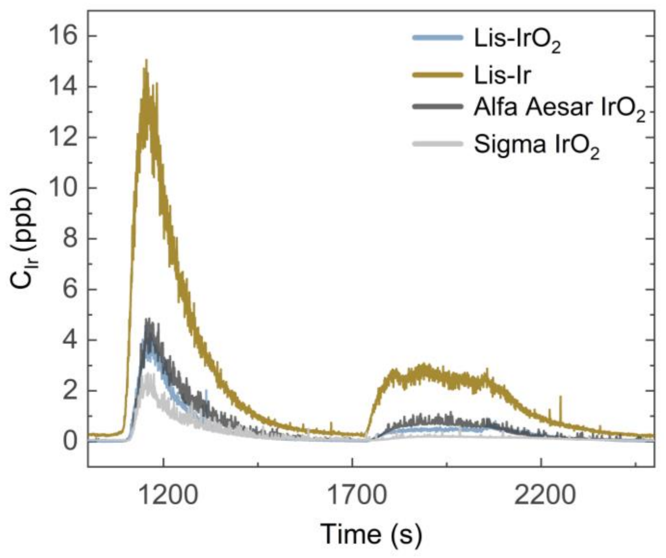

Figure S29. Dissolution stability analysis using CFC-ICP-MS: Second measurement of laser-synthesized catalysts and reference catalysts.

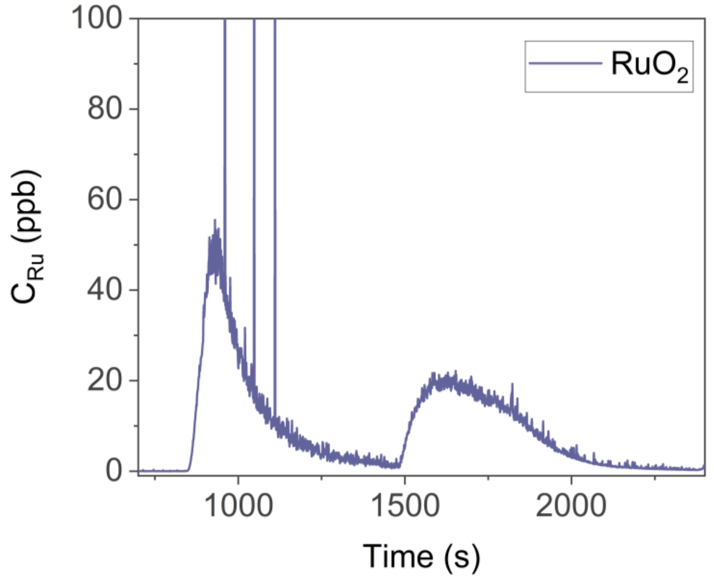

Figure S30. **Dissolution stability analysis using CFC-ICP-MS:** Second measurement of crystalline RuO_2

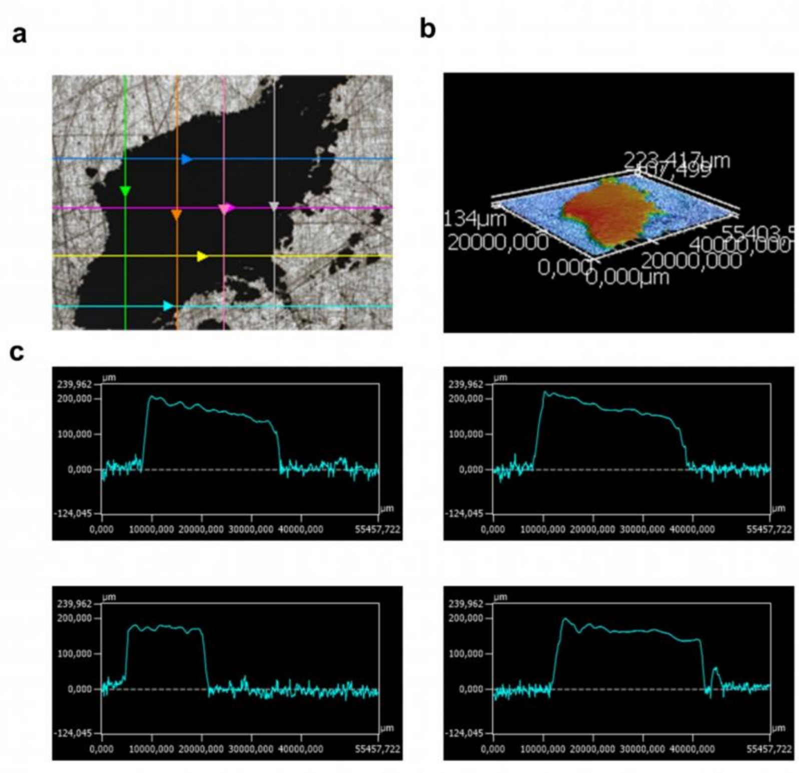

Figure S31. Laser scanning microscope measurements. a. Schematic representation of the plane measurement process used to determine the thickness of the powder layer on a titanium sheet. **b**. 3D visualization of the powder layer on the titanium sheet. **c**. Measurement reference using the titanium sheet as the baseline (0 µm height), with the powder

Table S1. Electrochemical surface area (ECSA) values determined by charge integration of the Ir^{3+}/Ir^{4+} redox peaks. The corresponding charge specific activity, turnover frequency (TOF), and mass specific activity measured at 1.53 V vs. RHE are also summarized for comparison.

Catalyst	Pseudo charge mC		Mass specific activity mA mg^{-1}		Charge specific activity mA mC^{-1}		ECSA m^2 g$_{Ir}^{-1}$		TOF s^{-1}
	value	error	value	error	value	error	value	error	value
Alfa Aesar	1.5985	0.092373	69.14049	14.5664	0.4239	0.0212	27.368	1.582	394.20
Sigma	0.0684	0.039783	13.87837	8.51046	1.9875	0.0994	1.172	0.681	1848.32
Lis-Ir	1.1238	0.040300	247.0672	0.97046	2.0925	0.0436	19.240	0.690	1945.95
Lis-IrO_2	1.1774	0.003213	350.5254	14.8484	2.8491	0.1057	20.158	0.055	2649.58

Note:

Determination of ECSA

The determination of electrochemical surface area (ECSA) is generally hard for iridium oxide-based systems, as techniques like H_{UPD} or CO stripping, established for platinum or other metals cannot be used. We follow a method described by GEIGER,[4] where the charge is calculated by integrating the measured CV curve between 0.4 V and 1.3 V vs RHE that reflects the oxidation process of $Ir^{3+} \rightarrow Ir^{4+}$. Division of the integrated area by the scanning rate of the CV yields the charge Q_{ox}. The ECSA in units of m^2/g is calculated by equation (1).

$$\boldsymbol{ECSA = \frac{Q_{ox}}{m_{cat} \cdot Q_{spec}}} \quad \textbf{(1)}$$

Q_{ox}: charge of $Ir^{3+} \rightarrow Ir^{4+}$ in C

m_{cat}: mass of catalyst on electrode in mg

Q_{spec}: specific charge in C/cm^2

For the specific charge of iridium oxide $596 \cdot 10^{-3} C/cm^2$ is assumed based on ZHAO et al.[5] For simplicity this value is assumed for all studied materials.

Determination of TOF

The turnover frequency (TOF) per active site is calculated from the current at 1.53 V vs RHE from the linear sweep voltammetry and calculated ECSA. The turnover number (TON) is calculated as an intermediate result (equation 2). For the TON, the OER in acidic media generating four electrons is considered:

$$2\ H_2O \rightarrow O_2 + 4\ H^+ + 4\ e^-$$

$$\boldsymbol{TON = \frac{j \cdot m_{cat}}{n \cdot F} \cdot N_A} \quad \textbf{(2)}$$

j: mass specific LSV current at 1.53 V vs RHE in mA/mg

n: number of transferred electrons per generated O_2

F: Faraday constant 96485.3 C/mol

N_A: Avogadro constant $6.022 \cdot 10^{23}$ 1/mol

$$\boldsymbol{TOF = \frac{TON}{\Gamma \cdot \mathrm{ESCA} \cdot m_{cat}}} \quad \textbf{(3)}$$

Γ: site density 1/m^2

For the site density an average value of $1 \cdot 10^{16}/\mathrm{m}^2$ is taken of as reported elsewhere.[6]

Table S2 The test conditions of compared catalysts for ICP-MS measurements.

Catalyst	LSV Ramping to	Hold	Electrolyte	Cell/Electrode
Lis-Ir	1.65 V_{RHE}	100 mA mg^{-1}_{Ir}	0.1M $HClO_4$	Channel flow cell/glassy carbon
Lis-IrO2	1.65 V_{RHE}	100 mA mg^{-1}_{Ir}	0.1M $HClO_4$	Channel flow cell/glassy carbon
RuO_2	1.55 V_{RHE}	100 mA mg^{-1}_{Ru}	0.1M $HClO_4$	Channel flow cell/glassy carbon
Sigma IrO_2	1.65 V_{RHE}	20 mA mg^{-1}_{Ir}	0.1M $HClO_4$	Channel flow cell/glassy carbon
Alfa Aesar IrO_2	1.65 V_{RHE}	100 mA mg^{-1}_{Ir}	0.1M $HClO_4$	Channel flow cell/glassy carbon
Umicore IrO_2/TiO_2	1.65 V_{RHE}	20 mA mg^{-1}_{Ir}	0.1M $HClO_4$	Scanning flow cell/glassy carbon
IrO_x(400°C)	5 mA cm^{-2}, ~1.62 V_{RHE}	N.A	0.1M $HClO_4$	Scanning flow cell/glassy carbon
IrO_2(600°C)	5 mA cm^{-2}, ~1.62 V_{RHE}	N.A	0.1M $HClO_4$	Scanning flow cell/glassy carbon
IrO_2(800°C)	5 mA cm^{-2}, ~1.62 V_{RHE}	N.A	0.1M $HClO_4$	Scanning flow cell/glassy carbon
IrO_2(film)	1.65 V_{RHE}	N.A	0.1M $HClO_4$	Scanning flow cell/glassy carbon
Ir(film)	1.65 V_{RHE}	N.A	0.1M $HClO_4$	Scanning flow cell/glassy carbon
IrOx(film)	1.65 V_{RHE}	N.A	0.1M $HClO_4$	Scanning flow cell/glassy carbon
Ba_2SrIrO_6	1.65 V_{RHE}	N.A	0.1M $HClO_4$	Scanning flow cell/glassy carbon

Table S3. Table of power parameters using CO_2 laser

Power / %	Power / W	Power / W cm^{-2}
0.03	0.024	32.71314
0.06	0.048	65.42629
0.1	0.08	109.04382
0.2	0.16	218.08763
0.4	0.32	436.17526
1	0.8	1090.43816
1.5	1.2	1635.65724
2	1.6	2180.87631

**The maximum power of CO_2 laser is 80 W and beam diameter is 305.71 μm for calculation.*

Table S4. Table of power parameters using fiber laser

Power / %	Power / W	Power / W cm^{-2}
0.1	0.02	167.72063
0.2	0.04	335.44126
0.5	0.1	838.60315
0.8	0.16	1341.76503
1	0.2	1677.20629
1.2	0.24	2012.64755
1.5	0.3	2515.80944
2	0.4	3354.41258
3	0.6	5031.61888

**The maximum power of fiber laser is 20 W and beam diameter is 123.25μm for calculation.*

Reference

(1) Pecharsky, V. K.; Zavalij, P. Y. *Fundamentals of Powder Diffraction and Structural Characterization of Materials Fundamentals of Powder Diffraction and Structural Characterization of Materials 1 2 3 Second Edition Second Edition*.

(2) Hartig-Weiss, A.; Tovini, M. F.; Gasteiger, H. A.; El-Sayed, H. A. OER Catalyst Durability Tests Using the Rotating Disk Electrode Technique: The Reason Why This Leads to Erroneous Conclusions. *ACS Appl. Energy Mater.* **2020**, *3* (11), 10323–10327. https://doi.org/10.1021/acsaem.0c01944.

(3) Lazaridis, T.; Stühmeier, B. M.; Gasteiger, H. A.; El-Sayed, H. A. Capabilities and Limitations of Rotating Disk Electrodes versus Membrane Electrode Assemblies in the Investigation of Electrocatalysts. *Nat. Catal.* **2022**, *5* (5), 363–373. https://doi.org/10.1038/s41929-022-00776-5.

(4) Simon Geiger. Stability Investigations of Iridium-Based Catalysts towards Acidic Water Splitting, 2018.

(5) Zhao, S.; Yu, H.; Maric, R.; Danilovic, N.; Capuano, C. B.; Ayers, K. E.; Mustain, W. E. Calculating the Electrochemically Active Surface Area of Iridium Oxide in Operating Proton Exchange Membrane Electrolyzers. *J. Electrochem. Soc.* **2015**, *162* (12), F1292–F1298. https://doi.org/10.1149/2.0211512jes.

(6) Karmakar, A.; Kundu, S. A Concise Perspective on the Effect of Interpreting the Double Layer Capacitance Data over the Intrinsic Evaluation Parameters in Oxygen Evolution Reaction. *Mater. Today Energy* **2023**, *33*, 101259. https://doi.org/10.1016/j.mtener.2023.101259.
